# “Eye-Conic” Spatial Transcriptomics Reveals the Layer-Specific Molecular Alterations in Corneas of Patients With Keratoconus

**DOI:** 10.1167/iovs.67.4.7

**Published:** 2026-04-03

**Authors:** Alicja Wysocka, Katarzyna Jaskiewicz-Rajewicz, Jakub Wozniak, Malgorzata Rydzanicz, Rafal Ploski, Monika Udziela, Jacek P. Szaflik, Marzena Gajecka

**Affiliations:** 1 Institute of Human Genetics, Polish Academy of Sciences, Poznan, Poland; 2 Initium BioData, Koscielna Wies, Poland; 3 Poznan University of Medical Sciences, Chair and Department of Genetics and Pharmaceutical Microbiology, Poznan, Poland; 4 Department of Medical Genetics, Medical University of Warsaw, Warsaw, Poland; 5 Department of Ophthalmology, Medical University of Warsaw, Warsaw, Poland; 6 SPKSO Ophthalmic University Hospital in Warsaw, Warsaw, Poland

**Keywords:** spatial transcriptomics (ST), cornea, keratoconus (KTCN), corneal layers, oxidative stress

## Abstract

**Purpose:**

Due to the structural complexity of the human cornea as well as the characteristic histopathological changes occurring in keratoconus (KTCN) cornea, there is a need to identify the associated cellular and molecular layer-specific alterations in the process of KTCN cone formation.

**Methods:**

The spatial transcriptomics (ST) approach was applied for KTCN and control corneas to investigate the layer-specific alterations. The data, organized into spatial clusters corresponding to corneal layers, were subjected to bioinformatic analyses, including pathways enrichment, spots deconvolution, and cell-cell communication network inference. Additionally, the outputs derived from our previous bulk RNA-seq experiment and immunofluorescence staining of corneal cryosections were evaluated.

**Results:**

The findings encompassing (i) deregulation of cell cycle, and peripheral accumulation of cell-cell junctions, pointing to a disrupted process of corneal epithelium (CE) renewal; (ii) upregulated extracellular matrix (ECM)-related pathways and enriched hallmark of epithelial-mesenchymal transition, affecting the stromal wound healing; (iii) activation of “Translation” and “Ribosome” pathways in the endothelium pointing to disrupted metabolic activity; and (iv) a unique cellular composition of KTCN cornea, with substantial stromal abnormalities. Furthermore, the downregulated expression of *MT2A* and *SAA1* genes, linked to cellular responses to stress and/or oxidative stress, was revealed across all KTCN corneal layers, corresponding to the identified underrepresentation of specific cell types. By comparing less- and more advanced KTCN phenotypes, the characteristic intercellular communication patterns were recognized.

**Conclusions:**

Findings regarding the corneal layers and the intercellular communication patterns in KTCN corneas allowed for further understanding of the cone formation mechanism.

Keratoconus (KTCN) is a degenerative, progressive disease of the cornea, characterized by its gradual thinning and steepening, which can lead to substantial impairment of visual acuity.^1^ KTCN has traditionally been considered non-inflammatory, which is now being disputed due to the emergence of reports of altered levels of cytokine, chemokine, and inflammatory mediators in patients with KTCN.^2^^,^^3^ Its onset typically occurs in adolescence and progresses to the third/fourth decade of life. The KTCN prevalence varies and depends on ethnicity and geographic location, with an estimate of 1:2000,^1^^,^^4^ and together with entities such as bullous keratopathy, Fuchs dystrophy, limbal stem cell deficiency, and keratitis, it constitutes one of the leading indications for corneal transplantation surgery (i.e. penetrating keratoplasty [PK]).^5^^,^^6^ However, the less invasive surgical treatments (e.g., corneal cross-linking [CXL] procedure) are usually considered before full corneal transplantation due to their high efficacy.^7^^,^^8^ Therefore, in the vast majority of KTCN experimental studies, blood, tears, buccal swab samples, or in vitro cell lines are being used, rather than focusing directly on the patients’ corneas, where the KTCN-specific histopathological changes may appear in each of the five layers. The corneal epithelial thinning, iron deposits in the basal layer, breaks in the Bowman's layer, reduction in the number of lamellae within the corneal stroma, and a significant decrease in endothelial cell density are being recognized as the most common features.^9^^–^^11^ However, a complete molecular characterization of the individual layers in the context of full-thickness corneas remains to be performed.

KTCN is a multifactorial disease, influenced by both genetics and environmental factors (eye rubbing, sun exposure, and male sex).^12^^–^^14^ Many attempts toward unraveling the genetic background of this disease have been undertaken, ranging from basic molecular techniques to high-throughput technologies paired with computational and statistical methods.^15^ Until now, at the transcriptomic level, the microarrays,^16^ “bulk” RNA-seq,^17^ and single-cell RNA-seq (scRNA-seq)^18^^,^^19^ techniques have been used. The assessment of the transcriptome at the single cell level (scRNA-seq) focuses mainly on the characterization of the cell types and subtypes in terms of gene expression in the context of disease or to unravel the physiological conditions.^18^^–^^21^ When using analyses at the single-cell level, location information is lost, which is extremely important in the case of a whole tissue or an organ. The full-thickness cornea, as a complex tissue, requires an approach such as spatial transcriptomics (ST)^22^ that will allow the characterization of individual layers with location preservation to discuss the process of KTCN cone formation. To date, in the only published study regarding the ST of the KTCN cornea, the involvement of the inflammatory responses in the KTCN progression in two, central and peripheral, corneal regions, but not corneal layers, has been investigated.^23^

In our previous study, the consolidation of the identified molecular, morphological, and clinical features enabled a discussion of the impaired wound healing process in KTCN, and we proposed the mechanism of corneal epithelium (CE) remodeling during the course of the KTCN.^24^ However, that model is limited to the CE, and the knowledge gap in the aspects of mechanistic abnormalities in the residual corneal layers in KTCN corneas remains to be filled in.

Here, we hypothesized that, in KTCN, the altered structure of the corneas and layer-specific histological changes are accompanied by unique molecular and cellular alterations. By identifying differentially expressed genes (DEGs) in the corneal layers, their corresponding molecular pathways, and characterizing the cellular composition in individual layers as well as alterations in cell-cell interactions, we aimed to enhance the current basic knowledge of KTCN and propose a detailed model of the mechanism underlying the KTCN cone formation.

## Methods

### Human Corneas

Eight corneas were collected from eight unrelated Polish male patients with KTCN. Patients were examined at the Department of Ophthalmology at the Medical University of Warsaw, Poland, and qualified to undergo the PK procedure. All patients underwent ophthalmological examination, as previously described,^17^ including assessments of both uncorrected and best-corrected visual acuity, intraocular pressure (IOP), and epithelial thickness mapping using spectral-domain optical coherence tomography (SD-OCT). Information regarding age, sex, and medical history was collected from the ascertained patients with KTCN. The patient exclusion and inclusion criteria are listed in Supplementary Table S1. This study was approved by the Ethics Committee of the Poznan University of Medical Sciences (decisions no. 179/24 and 180/24). Written informed consent was obtained from every patient with KTCN following an explanation of the possible consequences of the study in accordance with the tenets of the Declaration of Helsinki research protocol.

Five normal/non-affected corneas collected postmortem from five deceased male donors provided by the Eversight Tissue Bank (Ann Arbor, MI, USA), meeting the inclusion and exclusion criteria listed in Supplementary Table S1, constituted the control material. The information regarding biological sex was obtained from the medical records.

### Corneal Samples’ Preparation and Quality Assessment

The full-thickness KTCN corneas (6.5–8.5 mm in diameter) were submersed in Optimal Cutting Temperature (O.C.T.) compound (Tissue Tek, Sakura) and immediately frozen on dry ice using Cryospray (Engelbrecht GmbH). The control corneas collected at the Eversight Tissue Bank were larger in diameter (11–12 mm) compared with the KTCN corneas.

The small fragments of each cornea were subjected to RNA extraction using RNA/DNA/Protein Purification Plus Micro Kit following the manufacturer's guidelines (Norgen Biotek, Thorold, Ontario, Canada). The RNA integrity number (RIN) was assessed using the Total RNA Nano/Total RNA Pico kit and Bioanalyzer 2100 (Agilent, Santa Clara, CA, USA; see Supplementary Table S2). Then, the O.C.T.-embedded corneas were cut on the cryostat (Leica, CM1860 UV) at −17°C, to obtain cryosections for further use. The sample size was limited by the design of the ST protocol and the technology used in this study.

### Optimization of Experimental Conditions of the Spatial Transcriptomics

The corneal cryosections were prepared, fixed, and stained according to the manufacturer’s protocol “*for Fresh Frozen*” approach (v.1, Visium, 10x Genomics; Pleasanton, CA, USA) with some in-house modifications. Briefly, the cryosections were directly mounted onto the chilled Visium Spatial Gene Expression Slide (10x Genomics) and stored at −80°C. Then, the samples were incubated at 37°C, fixed in ice-cold methanol, and subjected to staining using bluing buffer (Dako, Agilent), hematoxylin and eosin (H&E; hematoxylin solution, Mayer, and Eosin Y solution; Millipore Sigma) with minor duration adjustments. The optimal experimental conditions were provided through the selection of appropriate tissue cryosection thickness (10 µm) and permeabilization time (6 minutes; see details in Supplementary Methods S1).

### Spatial Transcriptomics Approach

To enhance the performance of the ST approach for each cornea, two or three 10-µm thick, consecutive cryosections of each cornea were placed in the Capture Areas of a Visium Slide. The slides with samples were stored at −80°C until tissue fixation and H&E staining, and immediately after microscopic imaging, they were subjected to further analysis. The permeabilization of corneal cryosections was performed with optimal incubation time. The cDNA synthesis and the following construction of spatial gene expression libraries were performed according to the manufacturer's protocol. The quality control of the obtained libraries was performed using a 2100 Bioanalyzer and High Sensitivity DNA chip (Agilent).

Pair-end sequencing was performed using a NovaSeq 6000 sequencer (Illumina, San Diego, CA, USA). Sequencing was performed with a recommended minimal value of 50,000 read pairs per one tissue-covered spot of the Capture Area.

### Spatial Transcriptomics Data Analysis and the Corneal Spatial Clusters

The raw FASTQ files and histology images were processed using the 10x Genomics Space Ranger software (version 2.1.0). Reads were aligned to the human Cell Ranger hg38 reference genome using STAR method. The ST data were pre-processed, including manual alignment of selected samples using the Loupe Browser (see Supplementary Fig. S1). Subsequently, the data were loaded into Seurat, normalized, and integrated. The graph-based clustering was applied at a resolution of 0.2, followed by embedding with UMAP. Based on clusters mapping onto the tissue images, the cryosections reflecting the inconsistent projection of the clustering pattern were excluded from analysis (see Supplementary Fig. S2). Next, the differential gene expression (DGE) analysis for each spatial cluster across the multiple samples was performed using the *limma voom* pipeline. The genes meeting the following criteria were considered differentially expressed: 0.5 ≤ Log2 fold change (FC) ≤−0.5, *P* value < 0.05, and false discovery rate (FDR) <0.01 (see Supplementary Methods S2). The identification of marker genes for each cluster was performed by differential expression analysis, comparing each cluster against the remaining dataset.

### The Comparative Approach, Encompassing the ST Data and the Bulk RNA-Seq Outputs

To examine the potential differences between ST and the bulk RNA-seq approach, the comparison of DEGs between those two studies was performed. Our previous bulk RNA-seq study involved 25 KTCN corneas, which were collected according to the same inclusion and exclusion criteria as in the current study, and 25 non-KTCN control corneas,^17^ derived from individuals ascertained from the same population. The genes with values of FDR <0.01, and FC >1.5 were incorporated into the comparison.^17^ The DEGs derived from the ST approach were the sum of DEGs for individual clusters. The relationship between those two studies was presented on the Venn diagram.^25^ Then, the correlation between Log2FC values of the DEGs of bulk RNA-seq and of the unclustered ST approach (without the division into clusters) was tested, to check the general correspondence between those datasets. To further verify if there is a dominance of DEGs of one of the corneal layers, in the bulk RNA-seq approach, the correlations with the DEGs of each separate spatial cluster were performed. The JASP software^26^ was used to perform the statistical analyses, including Pearson's correlation.

### The Enrichment Analyses for Each Recognized Corneal Spatial Cluster

The CAMERA method^27^ was used to perform Reactome^28^ Pathway Enrichment Analysis (PEA) for each recognized corneal cluster. The enrichment analysis of hallmarks was performed for all clusters separately, using the Hallmark Gene Set Collection.^29^ The pathways and hallmarks with the global FDR <0.01 were considered significantly enriched. The universal enrichment tool *clusterProfiler*^30^ was implemented for Kyoto Encyclopedia of Genes and Genomes (KEGG)^31^ PEA and visualization for defined clusters. We were only interested in pathways sharing at least two genes with our gene sets for individual clusters, using a *P* value cutoff of 0.05. The Benjamini-Hochberg (BH) method was applied for the *P* value adjustment.

### Reference-Free Cell-Type Deconvolution of Spatially Resolved Transcriptomic Data into the Topics

The *STdeconvolve* open-source R software package was used^32^ for the deconvolution approach (see the details in Supplementary Methods S3). Each spot is represented by a pie chart visualizing the proportion of cell types, that is, the topics. The 20 transcriptionally distinct topics were recognized based on the data generated in the Loupe Browser software. The annotation of cell types in the form of spots’ outlines was performed based on a list of marker genes for each of the evaluated corneal layers (Table 1). The top 10 dominant genes of each topic were subjected to Gene Ontology (GO) over-representation analysis regarding the biological process (BP) terms using the enrichGO() function of the *clusterProfiler* tool in RStudio software environment, applying the *P* value < 0.05 cutoff. The BH method was applied for the *P* value adjustment. The STRING analysis^33^ was applied to the top marker genes, specific for topics 9, 12, and 19.

**Table 1.** The List of Marker Genes for Each of the Evaluated Corneal Layers

Layer	Gene ID	Ensembl ID
Epithelium	*KRT12*	ENSG00000187242
Epithelium	*GJB6*	ENSG00000121742
Epithelium	*KRT3*	ENSG00000186442
Epithelium	*S100A14*	ENSG00000189334
Stroma	*LUM*	ENSG00000139329
Stroma	*KERA*	ENSG00000139330
Stroma	*COL12A1*	ENSG00000111799
Stroma	*DCN*	ENSG00000011465
Endothelium	*CA3*	ENSG00000164879
Endothelium	*RGS5*	ENSG00000143248
Endothelium	*SLC4A11*	ENSG00000088836
Endothelium	*MSMP*	ENSG00000215183

### The Reclustering of Corneal Spatial Cluster 2, the CE

The spots corresponding to CE (cluster 2) were extracted from the entire dataset and further preprocessed by reclustering using Seurat at a resolution of 0.2 and a PCA of 6, and visualized using UMAP. The list of marker genes for each recognized subcluster was generated using the Seurat FindAllMarkers() function. The differential expression was assessed with the Wilcoxon rank sum test, and *P* values were adjusted for multiple testing using the Bonferroni procedure. The genes expressed by at least 25% of cells, and meeting the following criteria: average Log2FC > 0.5; adjusted (adj.) *P* value < 0.05, were considered significant (see Supplementary Table S3; Supplementary Fig. S3). The GO over-representation analysis using the *clusterProfiler* R tool, applying the *P* value < 0.05 cutoff, and the BH adjustment method were performed to characterize the subclusters.

### The Differences in the Intercellular Interactions Between the Less and More Advanced KTCN Corneas

The KTCN corneas were categorized based on keratometric measurements’ values (Kf – flat keratometry and Ks – steep keratometry) as of less advanced (*n* = 3, Kf < 62.5 diopters [D] and Ks < 67.9 D) and of more advanced phenotype (*n* = 4, Kf ≥ 62.5 D and Ks > 67.9 D). The JASP Software was used for statistical analyses.^26^ The assumption of normality was assessed based on the Shapiro-Wilk test (*P* > 0.05). The differences between those two groups were assessed using *t*-test statistics. The CellChat^34^ software package was used for the cell-cell interaction visualization.

### Immunofluorescence Staining of Corneal Cryosections

The consecutive 10-µm thick corneal cryosections of selected KTCN and control corneas were subjected to immunofluorescence (IF) staining. Briefly, the tissue-covered slides were fixed in methanol, washed with PBST, and blocked in the blocking buffer. Then, the samples were incubated overnight at 4°C with anti-LUM antibody (Invitrogen, #PA5-76722, RRID: AB_2720449, at a concentration of 1:150), followed by washing with PBST and incubation with secondary anti-rabbit IgG antibody (Alexa Fluor 488 conjugate, Invitrogen, #A32790, RRID: AB_2762833, at a concentration of 1:200), at room temperature for 2 hours. Then, the samples were washed and incubated with propidium iodide for counterstaining nuclei, and the mounting medium was applied to the slide. The slide imaging was performed using a Leica STELLARIS Confocal Microscope (Leica Microsystems GmbH) with an objective magnification of 20×.

## Results

### Identification of the Five Corneal Spatial Clusters

**Figure 1.**
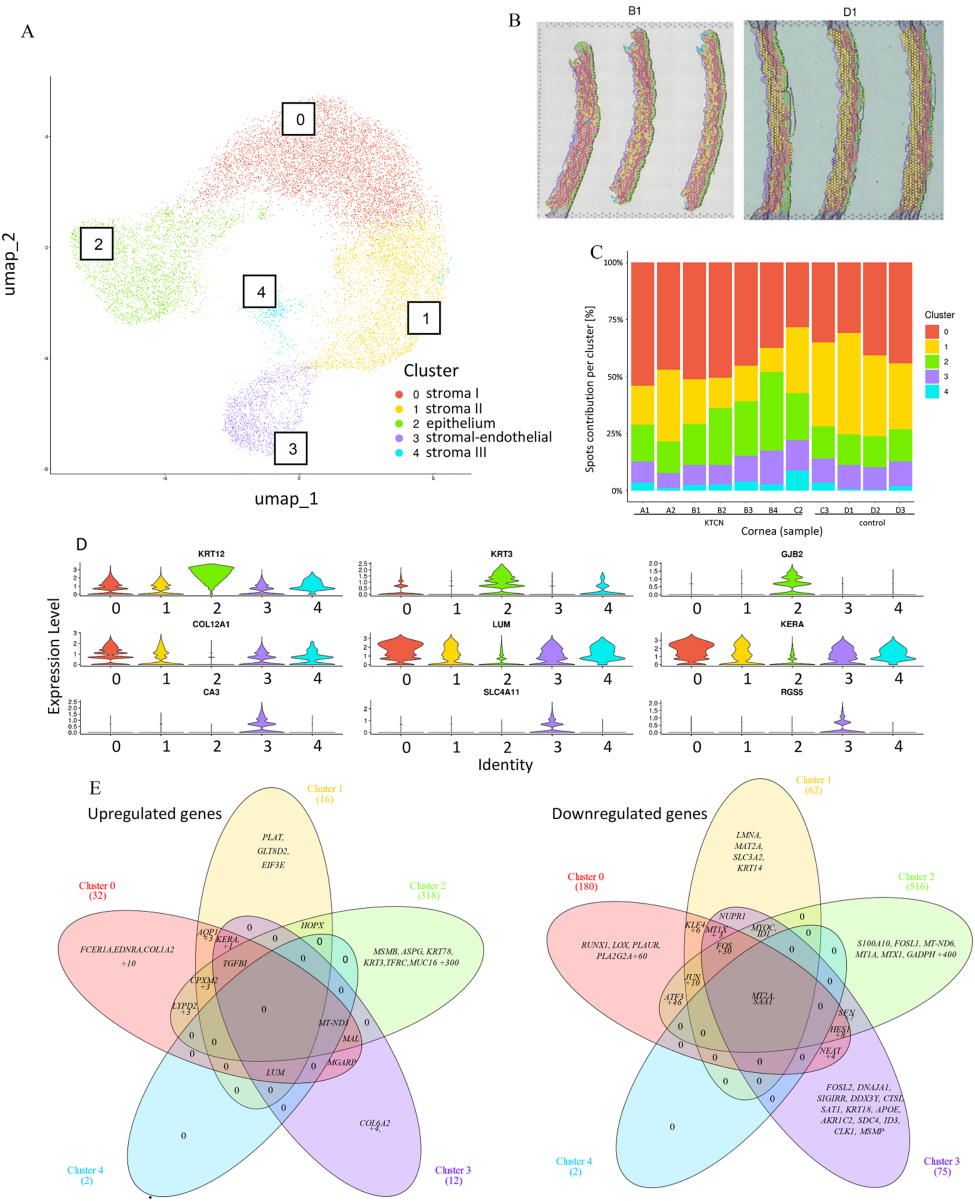
**Clustering results of ST data.** The preprocessing using Seurat pipeline, resolution of 0.2 (**A**) UMAP for spatial transcriptomics data, showing five clusters. (**B**) Based on the mapping of the clusters to the particular spots in 11 (7 KTCN samples and 4 control samples), we could infer the location within the corneal layers (**D1** – control sample and **B1** – KTCN sample), and therefore the approximate cell type: 0 – stroma I, 1 – stroma II, 2 – epithelium, 3 – stromal-endothelial, and 4 – stroma III. To facilitate direct visual comparison, panel **D1** was mirror-reflected so that both samples are presented in the same orientation. The original, non-modified orientation of **D1** is provided in Supplementary Figure S2. (**C**) The barplot shows the contribution of spots [%] of each of the clusters for each sample. Samples from **A1** to **C2** represent the KTCN group; **C3** to **D3** control group. (**D**) The expression of marker genes specific for epithelium (*KRT12*, *KRT3*, and *GJB2*), stroma (*COL12A1*, *LUM*, and *KERA*), and endothelium (*RGS5*, *CA3*, and *SLC4A11*) are presented at the violin plots. (**E**) The overlap analysis of upregulated (on the *left*), and downregulated (on the *right*) genes for each cluster. The two genes (*SAA1* and *MT2A*) were identified as downregulated regardless of the cluster. The diagrams were created using InteractiVenn (Heberle et al., 2015).^25^ Genes were considered significant meeting the following criteria 0.5 ≤ Log2FC ≤ −0.5, *P* value < 0.05, and false discovery rate (FDR) < 0.01.

Eight KTCN and five control corneas were subjected to the ST approach. The five clusters, 0, 1, 2, 3, and 4 (Fig. 1A), and spatial information in the form of a projection of their location within samples (Fig. 1B) were revealed upon the UMAP clustering. The spots’ contribution per cluster for each sample was checked (Fig. 1C; Supplementary Fig. S4). Cluster 2, based on the tissue mapping and high expression levels of epithelial marker genes, *KRT12*, *KRT3*, and *GJB2* (Fig. 1D), was defined as an epithelium (CE). In clusters 0, 1, 3, and 4, the increased expression levels of stroma marker genes, *COL12A1*, *LUM*, and *KERA,* were found; hence, we named clusters 0, 1, and 4 as stroma I, stroma II, and stroma III, respectively. Moreover, upon spatial projection, the location of clusters 0 and 1 corresponded to the division of corneal stroma into two regions, anterior and posterior. The spots forming cluster 3 presented high expression of endothelium-specific genes, *CA3*, *SLC4A11*, and *RGS5*. Because this cluster also showed high expression of stroma-specific genes, we classified it as a stromal-endothelial cluster. The other marker genes in each cluster are shown in the Supplementary Figure S5A.

### Transcriptional Abnormalities in KTCN Corneal Spatial Clusters

In the DGE analysis, comparing KTCN and non-affected corneas, performed separately for each corneal spatial cluster, 212 (180 down- and 32 upregulated) genes for cluster 0; 78 (62 down- and 16 upregulated) for cluster 1; 834 (516 down- and 318 upregulated) for cluster 2; 87 (75 down- and 12 upregulated) for cluster 3; and 4 (2 down- and 2 upregulated) for cluster 4 were revealed (see Supplementary Table S4; Supplementary Fig. S5B). The *MT2A* and *SAA1* genes were found to be downregulated in every cluster, based on the overlap analysis and visualization using a Venn diagram (Fig. 1E). Among the upregulated genes, the *LUM* expression was reported in all clusters except one (cluster 2), being restricted to the clusters with stromal features (or partially stromal). In the analysis of the top 10 deregulated genes (5 up- and 5 downregulated) per cluster, the alterations in the expression of *JUN*, *JUNB*, *FOS*, *FOSB*, and *ATF3* genes (see Supplementary Fig. S5C) were found.

### Comparative Analyses Including ST Data and Bulk RNA-Seq Results

The relationships between the findings of ST and bulk RNA-seq are illustrated in Supplementary Figure S6A; we found 116 genes with the same direction of change (36 upregulated and 80 downregulated in both), and 12 genes with different directions of change (Table 2). The *FOSL2* gene showed different expression patterns in clusters 2 and 3, up- and downregulated, respectively, in our ST approach. The positive correlation between the Log2FC values of DEGs from the bulk RNA-seq approach and the DEGs of unclustered ST data was found (Supplementary Fig. S6B). However, in comparison of bulk RNA-seq and DEGs of individual clusters, the strongest correlation was revealed for the CE (Supplementary Fig. S6C).

**Table 2.** The List of DEGs With Different Directions of Change in the Bulk RNA-Seq and the ST Data

	Direction of Changes	
Gene ID	Bulk RNA-Seq	ST Data
*NR1D1*	UP	DOWN
*HCAR3*	UP	DOWN
*NSA2*	UP	DOWN
*KRT18*	UP	DOWN
*SLCO4A1*	UP	DOWN
*COL6A2*	DOWN	UP
*MXRA5*	DOWN	UP
*LUM*	DOWN	UP
*CRABP2*	DOWN	UP
*GALNT5*	DOWN	UP
*WNT5A*	DOWN	UP
*GLT8D2*	DOWN	UP

### Pathway Enrichment Analysis in KTCN Corneal Layers

**Figure 2.**
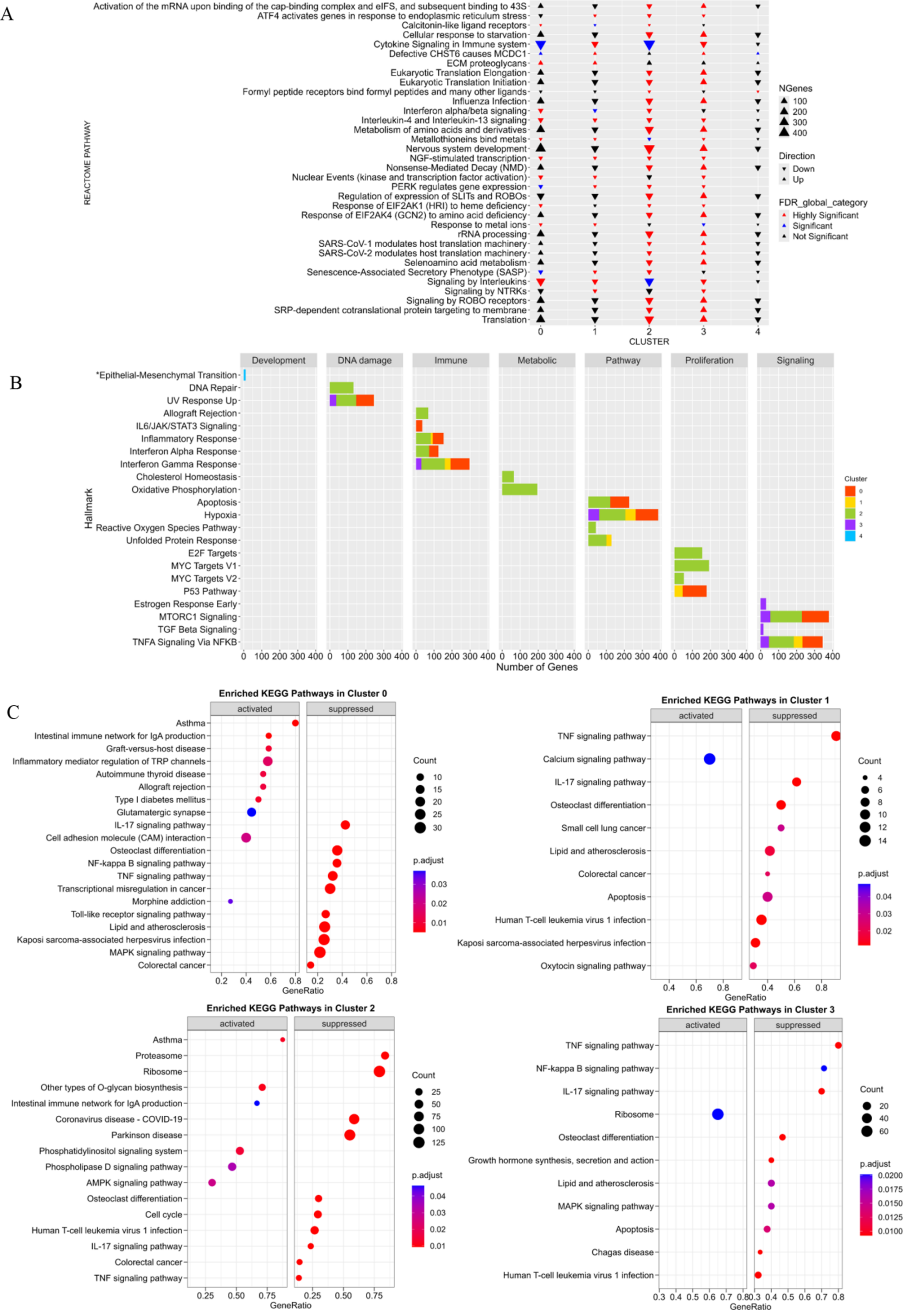
**The results of the pathway enrichment analyzes (PEA).** (**A**) The Reactome PEA. The pathways that were deregulated in more than one cluster are presented. The following classification was applied: 0.01 > FDR_global > 0 –, “Highly Significant”, 0.05 > FDR_global > 0.01 – “Significant”, 1 > FDR_global > 0.05 – “Not Significant”. *Arrows pointing up* represent the “Up” direction of change, and *arrows pointing down* – “Down” direction of change. (**B**) The barplots show hallmark gene sets, divided into clusters; all but one were downregulated; the one marked with an *asterisk* was upregulated. Only the hallmarks meeting the criteria of FDR_global < 0.01 are shown at the barplot. The color pattern was as follows: cluster 0 is *red*, cluster 1 is *yellow*, cluster 2 is *g*reen, cluster 3 is *violet*, and cluster 4 is *blue*. (**C**) The results of KEGG PEA for clusters 0, 1, 2, and 3. The significant pathways adjusted *P* value < 0.05. The majority of pathways were suppressed in the clusters.

The majority of the Reactome pathways were under-represented in the KTCN compared to non-affected corneas. The identified altered pathways were involved in the majority in the processes linked to Cellular Responses to Stimuli, Cell Cycle, or Signal Transduction. The 17 of 34 identified enriched pathways shared by at least two clusters (see Supplementary Table S5) showed a different direction of change for clusters 2 and 3 (Fig. 2A), belonging to process categories of “Cellular responses to stimuli” (*n* = 2), “Developmental biology” (*n* = 3), “Disease” (*n* = 3), “Metabolism” (*n* = 2), “Metabolism of proteins” (*n* = 5), and “Metabolism of RNA” (*n* = 2). Then, focusing on the unique Reactome pathways for individual clusters (see Supplementary Table S6) the highest number of under-represented pathways was found in cluster 2, corresponding to the CE, including the 29 related to the “Cell Cycle” and “DNA Replication”, which were this layer specific. Moreover, the “Keratan sulfate biosynthesis” and “Keratan sulfate/Keratin metabolism” pathways were recognized as specific for cluster 1, corresponding to stroma II.

In the gene set enrichment analysis of hallmarks, comparing KTCN and non-affected corneas, all but one of the altered sets were depleted (direction of change: “Down”; Fig. 2B). Most of them were characteristic of cluster 2 (CE), and included: “allograft rejection”, “cholesterol homeostasis”, “DNA repair”, “E2F targets”, “MYC targets v1”, “MYC targets v2”, “oxidative phosphorylation”, and “reactive oxygen species”. Only the “epithelial-mesenchymal transition” hallmark showed an “Up” direction of change, and at the same time, it was the only set found to be enriched in the analysis for cluster 4 (stroma III).

In the KEGG PEA, the general suppression of pathways was observed for clusters 0, 1, 2, and 3 (Fig. 2C). The cell cycle pathway disruption in cluster 2 was recognized, strongly emphasizing that this process was characteristic of KTCN CE. The “Ribosome” pathway activation was specific for the stromal-endothelial cluster (cluster 3). The IL-17 signaling pathway was suppressed in all four analyzed clusters.

### Spots Deconvolution Reveals the Differences in Cellular Composition Between KTCN and Control Corneas

**Figure 3.**
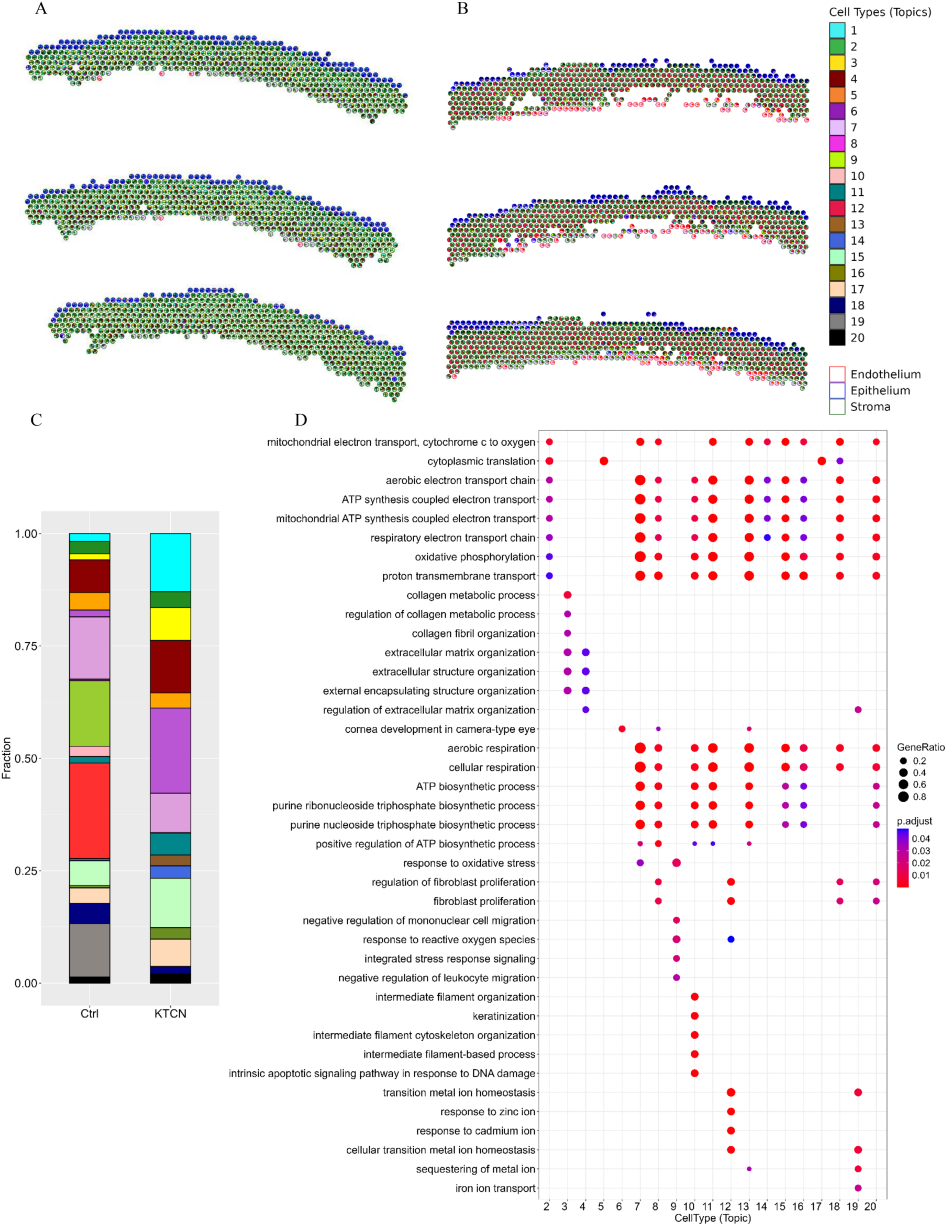
**The example of results of the ST deconvolution approach.** (**A**) KTCN cornea and (**B**) control cornea. The spots are deconvolved into cell types-topics, as pie plots. The epithelium, stroma, and endothelium cell types are projected as outlines of spots, based on the expression of marker genes. (**C**) The barplots of the cell proportions of cell types in non affected control (Ctrl) and KTCN corneas. The legend in the upper right corner is common for panels **A**, **B** and **C**. (**D**) The Gene Ontology (GO) over-representation analysis results. The results for top genes of each individual topic in the deconvolution analysis. The dot's size indicates the Gene Ratio, the colors represent the adjusted *P* values, with a gradient from *red* to *blue*, indicating higher to lower statistical significance. The Fisher exact testing and Benjamini-Hochberg correction were used.

The deconvolved ST data of KTCN and control corneal cryosections (Figs. 3A, 3B) differed within the corneal layers in the proportion of dominant topics, which reflects the cellular composition of the spot (Fig. 3C). Topic 6, substantially more abundant in KTCN, was found to be involved in “cornea development in camera-type eye” (GO:0061303).

Topics 9, 12, and 19 were not detected in KTCN corneas but were found to be specific for non-affected corneas. In the GO analysis of the top 10 genes for each topic, the topic 9 involved in the “response to oxidative stress” (GO:0006979), and “integrated stress response signaling” (GO:0140467), topic 12 responsible for responses to ions (GO:0010043 and GO:0046686) as well as “regulation of fibroblast proliferation” (GO:0048145), and topic 19 engaged in “regulation of extracellular matrix organization” (GO:1903053), and “iron ion transport” (GO:0006826) BPs were identified (Fig. 3D). In the STRING analysis of genes of topics 9, 12, and 19, the significant increase in interactions (protein-protein interaction [PPI] enrichment *P* value = 0.00081; Supplementary Fig. S7A) and enrichment in Ferritin complex and Transcription factor AP-1 complex were revealed (Supplementary Fig. S7B).

### The CE Reclustering Points to the Abnormal Cell Migration

**Figure 4.**
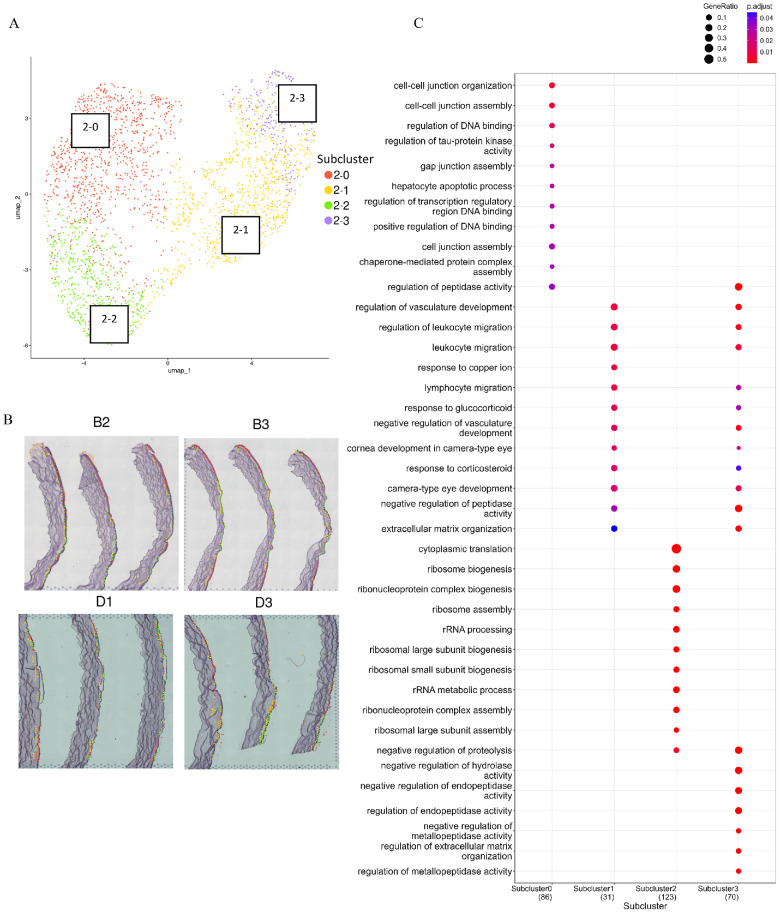
**The result of the re-clustering for the CE.** (**A**) The re-clustered corneal epithelium data of KTCN (*n* = 7) and control (*n* = 4) samples are presented at the UMAP. (**B**) The representative spots’ projection onto the tissue represent the specific subcluster organization around the corneal cone thinning, for the KTCN (**B2, B3**) and control (**D1, D3**) corneas. To facilitate direct visual comparison, panels **D1** and **D3** were mirror-reflected so that both samples are presented in the same orientation. The original, non-modified orientation of panels are provided in Supplementary Figure S2. (**C**) The results of GO analysis for each CE subcluster. The marker genes revealed for all CE subclusters were subjected to GO over-representation analysis regarding the biological process (BP) terms. The dot's size indicates the Gene Ratio, the colors represent the adjusted *P* values, with a gradient from *red* to *blue*, indicating higher to lower statistical significance. The Fisher exact testing and Benjamini-Hochberg correction were used.

As most of the identified pathways were disrupted in the CE, we performed reclustering of the spatial cluster 2, constituting the CE (Figs. 4A, 4B), and revealed the unique organization of subclusters 2-0, 2-1, 2-2, and 2-3 within this layer of KTCN cornea, with accumulation of subcluster 2-2 in the thinning area of the cone, and a more peripheral location of cluster 0. The GO terms related to “ribosome biogenesis” (GO:0042254), “rRNA metabolic process” (GO:0016072), and “cytoplasmic translation” (GO:0002181) were over-represented in subcluster 2-2. The “cell-cell junction organization” (GO:0045216) and “cell-cell junction assembly” (GO:0007043) terms were revealed for subcluster 2-0. For subcluster 2-3, the involvement in the “camera-type eye development” (GO:0043010) and “regulation of extracellular matrix organization” (GO:1903053) GO BP terms were detected (Fig. 4C).

### The Intercellular Communication in KTCN Corneas

**Figure 5.**
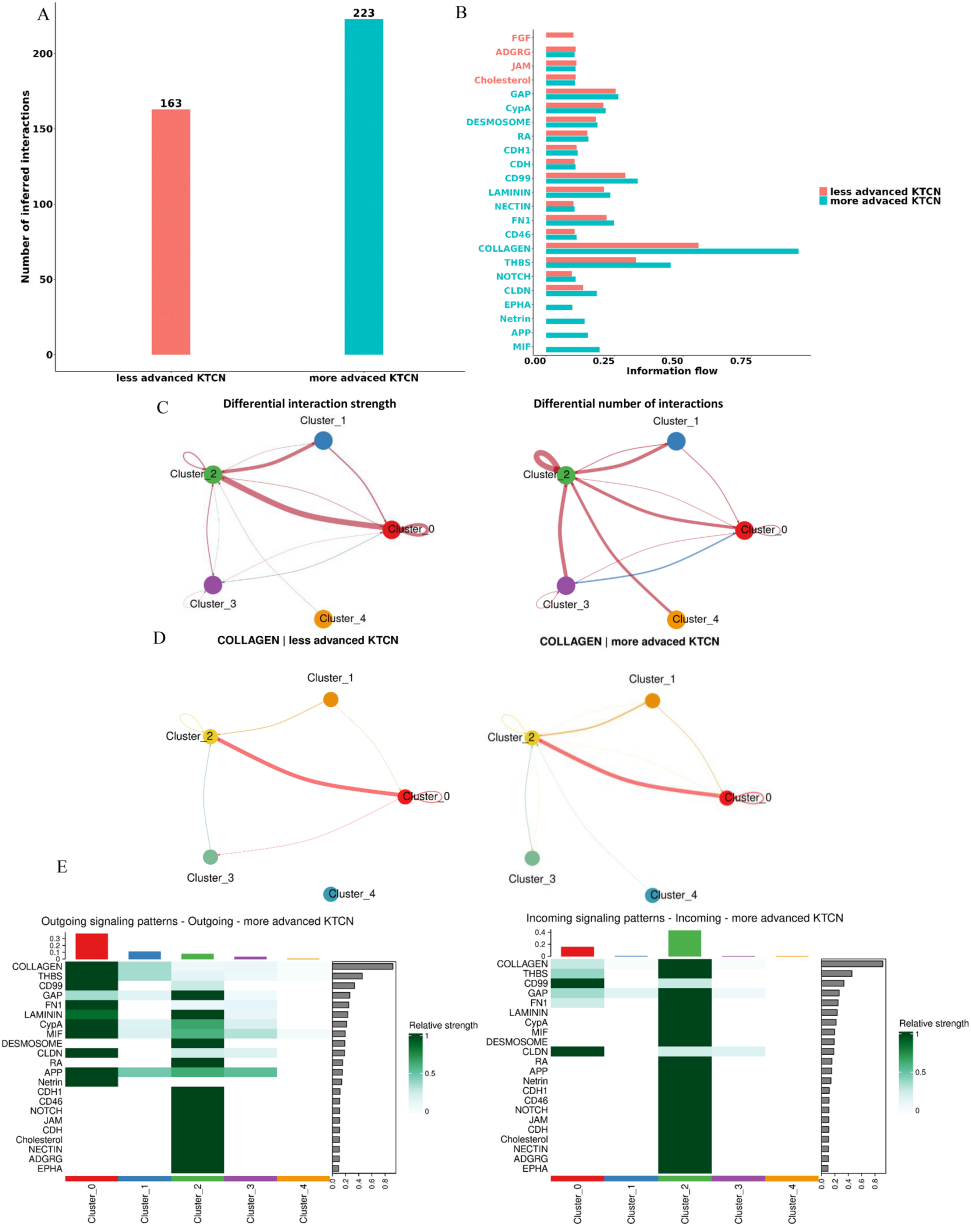
**The CellChat analysis of KTCN corneas.** (**A**) The overall interaction number for less and more advanced KTCN. (**B**) Information flow chart showing the total interaction strength of each pathway. The top signaling pathways colored *red* are more enriched in less advanced KTCN, the bottom ones colored with *blue* are more enriched in more advanced KTCN. (**C**) The differential number of interaction (*left*) and interaction strength (*right*) in the cell-cell communication network between two datasets. The *red* color indicates increased and the *blue* decreased signaling in the more advanced KTCN comparing to the less advanced. (**D**) The interactions for COLLAGEN pathway. (**E**) Outgoing (*left*) and incoming (*right*) signaling patterns of more advanced KTCN.

The recognized molecular and cellular alterations for specific layers, prompted to further explore the cell-cell communication networks between these layers. In the result of comparisons, the number of inferred interactions was 56 and 211, for control and KTCN corneas, respectively (see Supplementary Figs. S8A, S8B). The enrichment in the COLLAGEN pathway was more pronounced in the KTCN corneas. As the number and strength of interactions was higher in KTCN in comparison to control corneas, we decided to delve deeper into KTCN specific interactions, by comparing corneas with less (*n* = 3) and more advanced (*n* = 4) KTCN phenotypes. For less advanced corneas, the 163 interactions were inferred with the enrichment of FGF pathway, and for the more advanced corneas 223 interactions with the enrichment of MIF, COLLAGEN, and THBS pathways were found (Figs. 5A–C). The differences in the COLLAGEN pathway between those two groups involved the interactions between stroma I and endothelium in less advanced corneas, and the specific interactions of CE with endothelium in more advanced corneas (Figs. 5D, 5E; Supplementary Fig. S8C).

### The Altered Stroma Organization in KTCN Corneas

**Figure 6.**
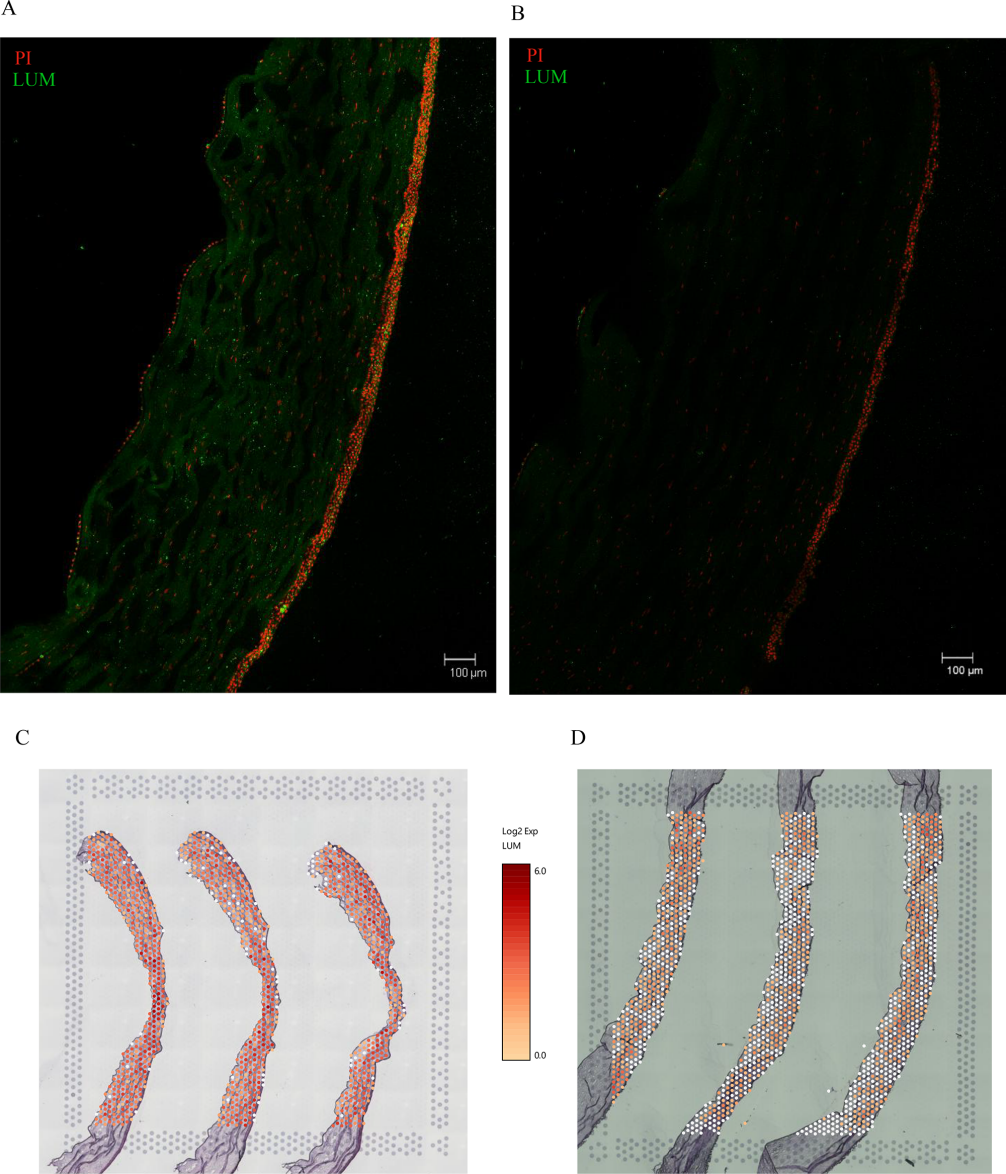
**The representative results of IF staining of 10** **-** **µm** **thick corneal cryosections.** The images capture under a confocal microscope, objective magnification of 20×. (**A**) KTCN cornea (**b3** sample); (**B**) control cornea (**d3** sample). For panels **A** and **B** there was one cryosection per sample. The red fluorescence signal (propidium iodide [PI]) indicates the nuclei, and the green fluorescent signal is specific for LUM. (**C, D**) The images from the Loupe Browser software visualizing the spatial gene expression of *LUM* gene on the adjacent corneal cryosections used for the ST approach of **C** KTCN cornea (**b3**), and (**D**) control cornea (**d3**), respectively. The comparison of the IF staining with the ST indicates the consistent change at the protein and transcriptomic levels of *LUM*. Based on the location and intensity of fluorescent signals, distinct stroma organization in the KTCN cornea was revealed. The differences in diameter of corneal samples between **C** KTCN and **D** control can be observed (as indicated in the Methodology section).

Based on the spatial clusters’ projection within the tissue, the altered distribution and disrupted proportion of the designated by us stroma I and stroma II, with the visible accumulation of spots representing the stroma I, were detected in the KTCN compared with control corneas. In non-affected corneas, the stroma I constituted approximately ⅓ of the whole stromal thickness, whereas in KTCN corneas it was approximately ⅔. Then, based on the upregulated expression of the *LUM* gene, specific for stroma-related clusters (0, 1, 3, and 4) as well as its distinct expression pattern in our previous bulk RNA-seq approach (as presented in Table 2), lumican was selected for further examination. By performing IF, the stromal-specific location of LUM protein was verified. Then, ST and IF outcomes were compared (Fig. 6). In the non-affected cornea, the stroma was visibly divided into (i) the anterior corneal stroma with a tight accumulation of lumican, and (ii) the posterior part with more dispersed lumican. In the KTCN corneal stroma, there was no such clear division.

### Mechanism of KTCN Cone Formation

**Figure 7.**
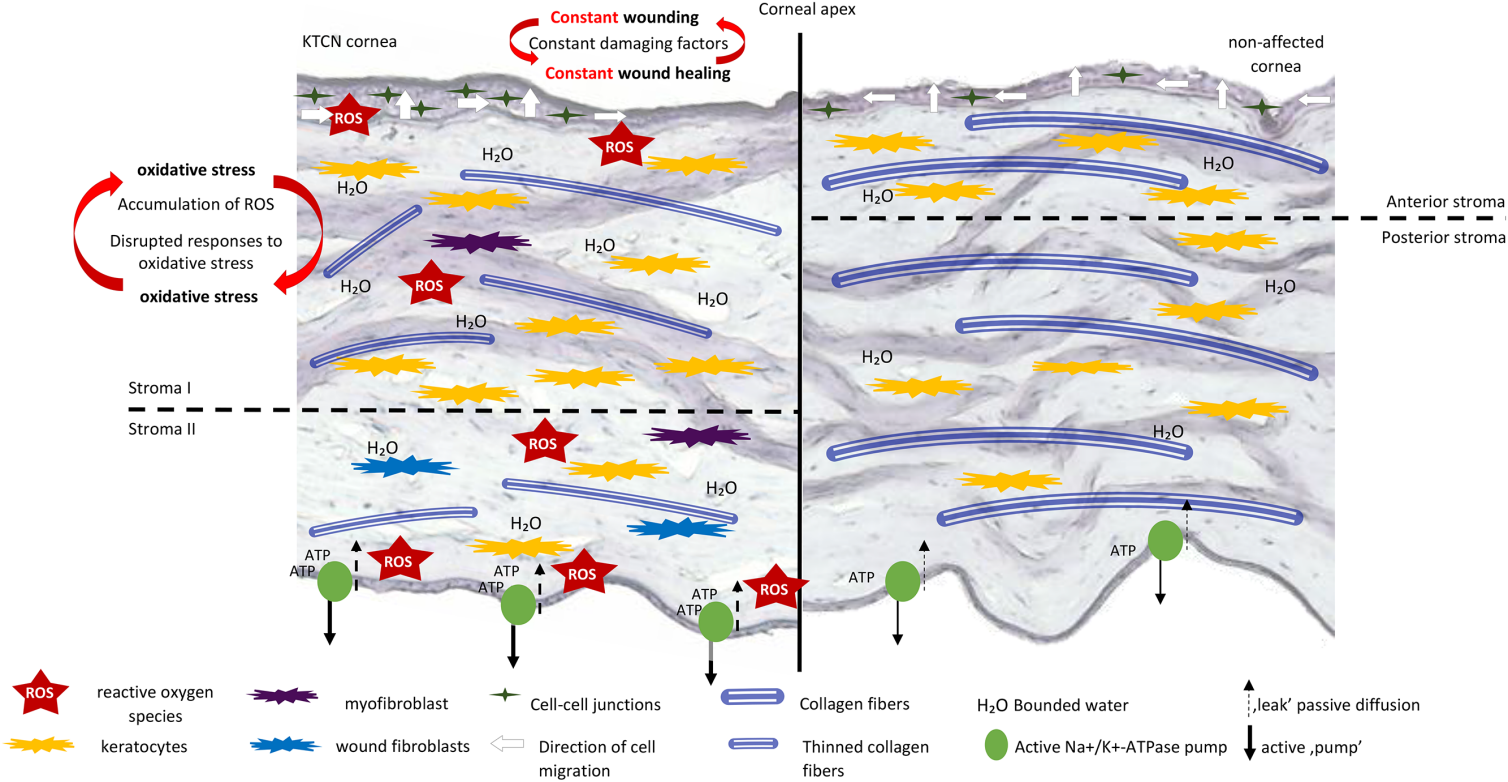
**The model of KTCN cone formation mechanism.** In the KTCN cornea (on the *left*), the accumulation of cell-cell junctions in corneal epithelium (CE) is marked with dark green stars at the periphery of KTCN cornea, and the abnormal direction of cells’ migration is indicated by *white arrows* from the periphery toward the center (corneal apex; as previously described in Jaskiewicz et al. 2023).^24^ In the control cornea, the cell-cell junctions are regularly distributed within the CE, and the correct direction of cells’ migration toward the center is marked with *white arrows* of the same size. The presence of phenotypically altered keratocytes in the stroma of the KTCN cornea was marked with colors: *yellow* indicates the normal keratocytes, *blue* indicates wounded fibroblasts, and *violet* indicates myofibroblasts. The structural differences in the distribution of stroma, with a disrupted ratio of stroma I to stroma II in the KTCN cornea and corresponding correct ratio of anterior to posterior parts in the non-affected cornea, are marked with the *dashed line*. The higher capacity of stroma I/anterior part of cornea to bind water is marked with the more abundant H₂O distribution. The increased activity of the endothelial “pump” function and “leak” passive diffusion of nutrients in the KTCN corneal endothelial cells is marked with the *arrows* (*bold* and *dashed*, respectively). The physiologically correct endothelial pump functions are marked with equally *thick arrows*, indicating the unaltered metabolic activity.

The identified molecular layer-specific abnormalities in the CE, stroma, and endothelium in KTCN cornea as well as common changes across the recognized spatial clusters, constitute the basis of our proposed KTCN cone formation mechanism. The recognized characteristic of KTCN cornea elements included in our model, (i) the downregulation of cell cycle processes in CE and peripheral accumulation of the subcluster related to cell-cell junctions, (ii) the distinct cellular and structural composition in stroma, and (iii) endothelial-specific abnormal metabolic activity, as well as (iv) deregulated responses to oxidative stress across the KTCN corneal layers, are shown in Figure 7, and described in detail in the Discussion section.

## Discussion

As opposed to the bulk RNA-seq approach, which delivers the average information regarding the gene expression across the mixture of cells in the sample,^17^^,^^24^ as well as previously applied scRNA-seq focusing on the gene expression in individual cells,^19^^,^^21^ in the ST approach, the spatial location is preserved; therefore, the gene expression outputs can be mapped in the tissue context. As we anticipated, the discrepancies in findings between the abovementioned studies were noted, with the ST enabling the separation of the obtained data in the layer-specific context, therefore pointing to locally narrowed KTCN-specific alterations in the cornea. The strongest correlation between bulk RNA-seq and CE cluster of the ST approach shows that in the bulk approach, the main information of the gene expression derives from CE, somehow masking the information from other layers. Moreover, the observed stromal upregulation of *COL6A2*, *LUM*, and *WNT5A* genes, previously reported as downregulated in our bulk RNA-seq approach,^17^ pointed to deregulation of fibril organization and cells’ proliferation, is an especially interesting result from this perspective.

By delving into the KTCN corneal layer-specific abnormalities, we have identified both the differentiating features as well as common traits among all layers, thus emphasizing the importance of assessing the tissue context in KTCN research.

### The Alterations in Epithelial Remodeling in KTCN Corneas

The initial phase of corneal wound healing, that is, cell migration, is highly dependent on the cell-cell adhesion, which, when too tight, prevents the cells from moving toward the injured area.^35^ The processes detected in our CE reclustering analysis for the subcluster 2-0, peripherally located in KTCN CE, and the detected high expression of genes *GJB2*, *GJB6* (coding gap junction proteins), *DSG1*, *JUP* (involved in the desmosome formation), and *CLDN4*, and *CLDN7* (coding components of tight junction strands) pointed to junctional complexes that are present at different depths of the CE. The gap junctions, which were highlighted in the GO analysis, are responsible for the mediation of intercellular communication and are apparent in the basal cell layer of the CE.^35^ The intercellular junctions are crucial for cell differentiation and maintenance of the intact CE. However, this junction over-representation was not specific for the subcluster 2-2, accumulated in the central region of the KTCN CE (the thinning area in the corneal apex). For this subcluster, the over-representation of the translation and ribosome-related processes as well as the high expression of a stem cell marker (*KRT14*) in corneal epithelial cells^36^ were revealed.

The general deregulation of the cell cycle in the CE, together with the accumulation of cell-cell junctions at its periphery, points to the disruption of the CE organization in the KTCN cornea, which influences the CE remodeling. These findings are in line with our previous multi-omic approach results, reduction of cells’ migration from the *middle* to the *central topographic* (the apex) *regions*.^24^

Although at the stage of the ST permeabilization, the highest fluorescent signal was derived from the epithelial corneal layer, and we found that most of the deregulated genes in the KTCN cornea were involved in CE-specific molecular pathways, this does not mean that the majority/most important KTCN-specific processes or mechanisms are restricted to the CE. Below, we discuss equally important abnormalities identified in the other corneal layers.

### The Signatures of Structural and Transcriptional Stromal Abnormalities

The anterior part of the stroma affected by high oxygen tension, has a high capacity to bind water, whereas the posterior part, less affected by evaporation, loosely bound water, which is required for transport across the endothelium via the metabolic pumps.^37^ In the healthy cornea, the anterior part of the stroma constitutes approximately ⅓ of stromal thickness, whereas the posterior part comprises the remaining ⅔.^37^ The imbalance detected in our study, with the thicker stroma I in KTCN compared to the non-affected anterior part (in control individuals), may suggest the higher water-binding capacity of the KTCN cornea.^37^ In stroma II, the enrichment of extracellular matrix (ECM)-related pathways was recognized in KTCN corneas.

In the process of repair/healing of a corneal wound resulting from injury or disease, two phases can be distinguished, which are accompanied by the phenotypically altered keratocytes. Present in the initial phase – hypercellular myofibroblasts, which then can transform either into wound fibroblasts that produce high levels of proteoglycans or myofibroblasts producing a low level of ECM.^38^ In our study, the altered molecular pathways in stroma II point to its involvement in the second phase of stromal wound healing. Delving into this aspect, based on the identified upregulated hallmark of epithelial-mesenchymal transition corresponding to the keratocyte trans-differentiation to myofibroblasts, in stroma III in our study, we noticed signs of the initial stage of wound healing in the stroma, pointing to a continuous process of stromal disruption and healing.^39^

The over-represented pathways of the responses to ions, response to oxidative stress, regulation of fibroblast proliferation, and regulation of ECM organization in topics 9, 12, and 19, which are absent/substantially underrepresented in KTCN cornea, point to disruption of the normal physiological function of the corneal stroma. The marker genes for the topics 12 and 19 include the *MT2A* and *SAA1*, which are in line with their downregulation in KTCN corneas identified in the DGE analysis.

*LUM*, which in our study was noted as upregulated in all but one cluster (cluster 2) in KTCN corneas, is a gene coding for a proteoglycan, and has been previously reported as upregulated in the corneal stroma wound healing.^40^ Lumican colocalizes with fibrillar collagens in the corneal stroma, maintains the strict collagen architecture of the cornea, regulates the collagen fiber diameter, and has an inhibitory effect on the rate of fibrillogenesis as well as organization of collagen fibrous network.^41^^–^^45^ Therefore, we conclude that the visible differences in LUM distribution in the IF images are related to the abnormalities in collagen fibril organization, what might influence the corneal stiffness, and in consequence to the changed/altered shape of KTCN cornea.^46^

The characteristic for KTCN corneas activation of interactions with SDC1 (syndecan), THBS4 (thrombospondin), which are involved in the ECM organization, mediating ECM fiber alignment^47^^,^^48^ and wound healing,^49^ respectively, points to the active process of ECM remodeling in the KTCN cornea in which mainly stroma I and stroma II are involved.

### The Endothelial Layer in the KTCN Cone Formation Model

The corneal endothelium is responsible for the maintenance of corneal transparency by relative stromal dehydration, along with the correct alignment of stromal collagen.^50^^,^^51^ In our study, the opposite direction of changes in the Reactome pathway analysis was revealed in KTCN corneas; the selected pathways, depleted in the CE, are enriched in the KTCN corneal endothelium. The translation-related pathways emerged as the main process in this comparison, together with the activation of the “Ribosome” KEGG pathway, indicating the potentially increased protein synthesis in the endothelium of KTCN corneas. Because the major types of proteins produced by the endothelial cells are: enzymes, structural proteins, and those constituting the ECM,^52^ the recognized upregulation of translation can potentially be directed toward the support of the maintenance of proper stromal structure as well as nourishment and hydration.

Moreover, the *MT-ND3* and *ATP5MD* genes, upregulated in corneal endothelium, code the components of mitochondrial complexes of the respiratory chain, involved in the ATP production, utilized for maintenance of correct endothelial “pump” function,^53^ hence, the proper corneal stroma hydration that may be disrupted in the KTCN cornea. Conversely, several components of the unfolded protein response pathway, including ATF6-mediated chaperone gene activation, were significantly depleted in the endothelium. This downregulation may reflect an impairment of endoplasmic reticulum (ER) stress signaling, potentially compromising the cell's capacity to manage proteotoxic stress and maintain protein homeostasis.

### Disrupted Oxidative Stress Responses are Common for all Corneal Layers in KTCN Corneas

Among the detected DEGs, the *MT2A* as well as the *SAA1* genes were found to be downregulated in all five recognized clusters. The *MT2A* is a member of the metallothionein (MT) family of genes, which encode the proteins that act as antioxidants and are important in the homeostatic control of metal in the cell. *SAA1* encodes a member of the serum amyloid A family of apolipoproteins, and it is induced during the acute-phase response to infection or during injury by proinflammatory cytokines, indicating that those processes are impaired in KTCN.^54^ Based on the identified across all corneal layers, both the oxidative nature of *MT2A* as well as the general reduction of antioxidant potential/stress response, including oxidative stress in the KTCN cornea, we conclude that the accumulation of reactive oxygen species (ROS) influences the deregulation of regenerative processes in the cornea.

The *JUN*, *JUNB*, *FOS*, and *FOSB*, downregulated in the majority of KTCN corneal clusters, are subunits of the dimeric activator protein-1 transcription factor complex (AP-1). That is in line with the absence of topics 9, 12, and 19 in KTCN corneas, for which their marker genes are involved in the transcription factor AP-1 complex. The AP-1 was initially described as a DNA-binding protein that recognized a DNA element found in the enhancer region of, among others, the aforementioned *MT2A.*^55^ Those AP-1 subunits are known to regulate a wide range of processes, such as cell proliferation, differentiation, and apoptosis.^56^ There are reports on the long-term chronic effects of oxidative stress on downregulation of AP-1 activity. Moreover, in the transcriptomic data analysis of two distinct patient populations: Middle Eastern and African American, a significant decrease in the gene expression levels of other AP-1 subunits, *ATF3*, *FOSL1*, *PPP1R15A*, and *PTGS2* in KTCN corneas were found,^57^ which is concordant with our results.

### Study Limitations

The growing number of alternative treatments and mitigation of the symptoms in patients with KTCN are major limitations to the number of patients ascertained and full-thickness corneas derived for this type of project.

The ST approach “*for Fresh Frozen*” (v.1) was applied, with some technical limitations: (i) the tissue's cryosections were applied directly onto the 6.5 × 6.5 mm Capture Area of the slide, and once mounted, could not be repositioned, which required precise operation, (ii) the resolution of the applied ST version is less precise than a single-cell level, because the 55 µm spots on the slide can capture multiple cells. However, we have overcome this issue using the deconvolution/bioinformatic approaches. Despite various immune-related pathways being noted as dysregulated in this study, the resolution of the applied assay, as mentioned above, does not allow the identification/characterization of single macrophages, DCs, T and B cells; this aspect should be further investigated using, for example, the single cell-based approaches.

As the male sex is a risk factor of KTCN, and the prevalence differs depending on ethnicity, in our study, we included the Caucasian male individuals only.

## Conclusions

The recognized across all KTCN corneal layers, deregulation of response to oxidative stress, corresponding to the unique cellular composition of KTCN cornea, and the accumulation of ROS originated from internal and external sources (damaging factors such as eye rubbing or UV radiation/light) influence the regulation of regenerative processes in the cornea. The abundance of ROS may disrupt the signal transduction and impair the immune response of cells, which further highlights the pivotal role of oxidative stress in the pathophysiology of KTCN. The model of full-thickness KTCN cornea cone formation, considering the identified cellular and molecular layer-specific alterations indicated in this study, especially CE wound healing, and the substantial alterations in stroma and endothelium, supplemented with the characteristic intercellular communication patterns, fills the knowledge gap regarding the mechanistic abnormalities in the full-thickness KTCN cornea, and supports the improvement of experimental models for further KTCN research.

## Supplementary Material

Supplement 1

Supplement 2

Supplement 3
