# Supplementary material for: “Eye-Conic” Spatial Transcriptomics Reveals the Layer-Specific Molecular Alterations in Corneas of Patients With Keratoconus
Source: Invest Ophthalmol Vis Sci. 2026 Apr 3;67(4):7. doi: 10.1167/iovs.67.4.7 (PMC13056006; doi:10.1167/iovs.67.4.7)
Supplement: Supplement 1 [file iovs-67-4-7_s001.pdf]

SUPPLEMENTARY MATERIALS

'Eye-conic' spatial transcriptomics reveals the layer-specific molecular alterations in corneas of patients with keratoconus

SUPPLEMENTARY METHODS

Supplementary Methods S1. Optimization of experimental conditions of the spatial transcriptomics

The first requirement for achieving reliable results is a suitable thickness of the tissue cryosection. We examined three different thicknesses of corneal cryosections, 7 µm, 10 µm, and 12 µm. The O.C.T. embedded corneas were mounted on specimen discs (Leica Biosystems) and equilibrated to the cryostats' (Leica, CM1860 UV) chamber temperature (-17°C) for 30 minutes before sectioning. The excess O.C.T. compound was trimmed off with a razor blade. The cryosections were directly mounted onto the chilled Visium Spatial Gene Expression Slide (10x Genomics) and stored at -80°C. Then, the samples were incubated at 37°C, fixed in ice-cold methanol, and, without the addition of isopropanol, subjected to staining using bluing buffer (Dako, Agilent), hematoxylin and eosin (H&E) (Hematoxylin Solution, Mayer, and Eosin Y solution, Millipore Sigma) with minor duration adjustments. The Capture Areas were captured individually under light microscopy (Leica Microsystems, DM5500). Three different thicknesses of corneal cryosections, 7 µm, 10 µm, and 12 µm, were assessed based on H&E staining and light microscopy (Leica, DM5500). It was observed that 7µm cryosections were challenging to cut on cryotome, and too thin to manipulate. On the contrary, 12µm sections were simple to obtain, the corneal epithelium was intact, although it was difficult to obtain a good-quality microscopic image, due to discrepancies in z-axis positions. Based on the technical considerations in obtaining the intact cryosections, the 10 µm thickness was selected as suitable.

After the cryosection thickness establishment, prior to the actual Gene Expression workflow, the Tissue Optimization (10x Genomics), recommended by the manufacturer, was performed. Choosing the permeabilization time suitable for specific tissue types is a crucial step in releasing mRNA, which then binds to capture probes on the slide. The appropriate tissue permeabilization time was chosen by comparing 3 different times, 3 min, 6 min, and 12 min, using the Tissue Optimization Slide (10x Genomics, PN3000394) and the Tissue Optimization Kit (10x Genomics). Positive (Universal Mouse Reference RNA, QS0640, Thermo Fisher Scientific) and negative controls were included in the tissue optimization step. Considering the fact, that if the fluorescent signal is the same at the two time points, the longer permeabilization time is recommended to apply, the time of 6 minutes was selected.

Supplementary Methods S2. The spatial transcriptomic data analysis and the corneal spatial clusters

The raw FASTQ files and histology images were processed using the 10x Genomics Space Ranger software (version 2.1.0). Reads were aligned to the human Cell Ranger hg38 reference genome (available at https://cf.10xgenomics.com/supp/spatial-exp/refdata-gex-GRCh38-2020-A.tar.gz) using the STAR method. Following initial quality assessment, the ST data underwent pre-processing, including manual alignment of selected samples using Loupe Browser. Briefly, the manual alignment using the Visium Image Alignment function in Loupe Browser was used for sample D2 to exclude spots of non- corneal tissue fragments from further analysis spots that belong to the tissue region that contain fragment of trabecular meshwork based on the morphological assessment after H&E staining as well as the increased expression of *MYOC* (Supplementary Fig. S3). Subsequently, the data were loaded into Seurat as separate objects. Quality control included the removal of barcodes with zero counts. Normalization was performed using the SCTransform() function, and data were integrated using canonical correlation analysis (CCA). Integration was based on 2000 highly variable features selected across samples using SelectIntegrationFeatures(), followed by identification of integration anchors using FindIntegrationAnchors(). The graph-based clustering was applied at a resolution of 0.2, followed by embedding with Uniform manifold approximation and projection (UMAP). Each of the five generated corneal spatial clusters represents a group of spots that share a similar pattern of gene expression and reflect the underlying organization of tissue, possibly coinciding with different cell

types. Their identification was performed by assessing the expression of the known marker genes specific for the particular corneal cell types corresponding to corneal layers in individual clusters. Based on clusters mapping onto the tissue images, the cryosections reflecting the inconsistent projection of clustering pattern were excluded from analysis (Supplementary Fig. S4) we have noticed that 2 of cryosections in 2 independent samples, all well as samples C4 and D4 show different clustering pattern projection onto the tissue, and we decided to exclude them from further analysis. Next, the differentially expressed genes (DEGs) analysis for each spatial cluster across the multiple samples was performed using the *limma voom* pipeline, with a cut-off of min.count = 10 for each sample, and min.total.count = 15 in all samples. The following criteria were applied: $0.5 \leq Log2FC \leq -0.5$, p-value < 0.05, and false discovery rate (FDR) ≤ 0.01.

Supplementary Methods S3. Reference-free cell-type deconvolution of spatially resolved transcriptomics data into the topics.

The *STdeconvolve* open-source R software package was used for the deconvolution approach. The data from the samples was aggregated into a single feature-barcode matrix using the *spaceranger aggr* pipeline. The low-quality spots and genes expressed at low levels were filtered out before modelling, retaining genes expressed in at least 1% of spots and spots with at least 100 detected transcripts. Each spot is represented by a pie chart visualizing the proportion of cell types, i.e., the topics. The 20 transcriptionally distinct topics were recognized based on the data generated in the Loupe Browser software. The annotation of cell types in the form of spots' outlines was performed based on a list of marker genes for each of the evaluated corneal layers (Table 2).

The top 10 dominant genes of each topic were selected based on the highest values in the β matrix, e.i, the distribution of genes in topics. Next, they were subjected to GO over-representation analysis regarding the BP terms using the enrichGO() function of the *clusterProfiler* tool in RStudio, applying the p-value <0.05 cut-off. The BH method was applied for the p-value adjustment.

SUPPLEMENTARY FIGURES

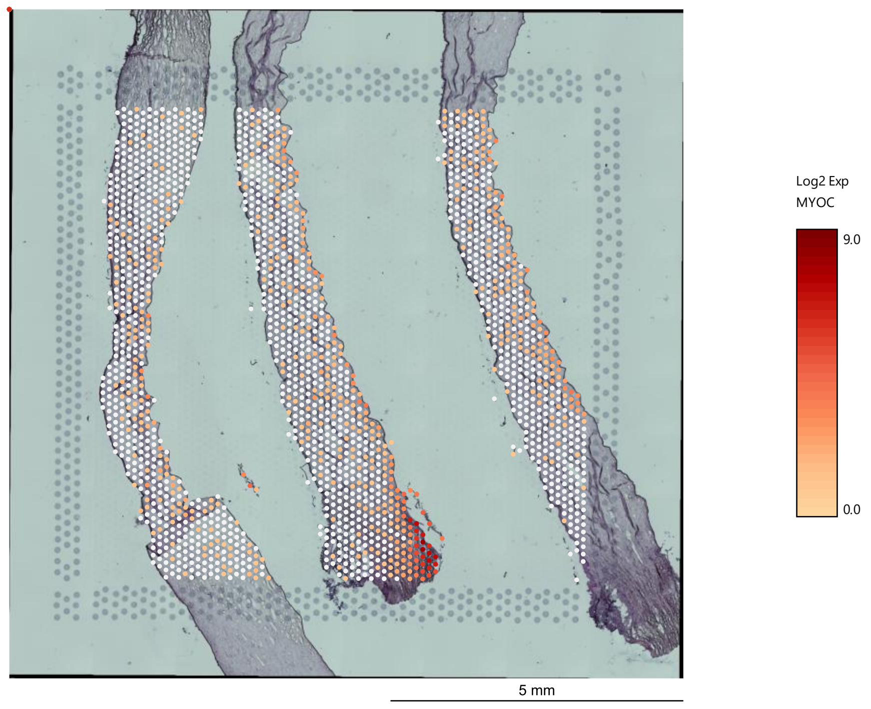

Supplementary Fig. S1. The expression of *MYOC* gene, characteristic for trabecular meshwork. Sample D2 was subjected to manual alignment using the Visium Image Alignment function in Loupe Browser software (v.8.0.0) to exclude spots that belong to the tissue region to exclude spots that belong to the tissue region containing fragments of trabecular meshwork, based on the morphological assessment after H&E staining as well as the increased expression of *MYOC*.

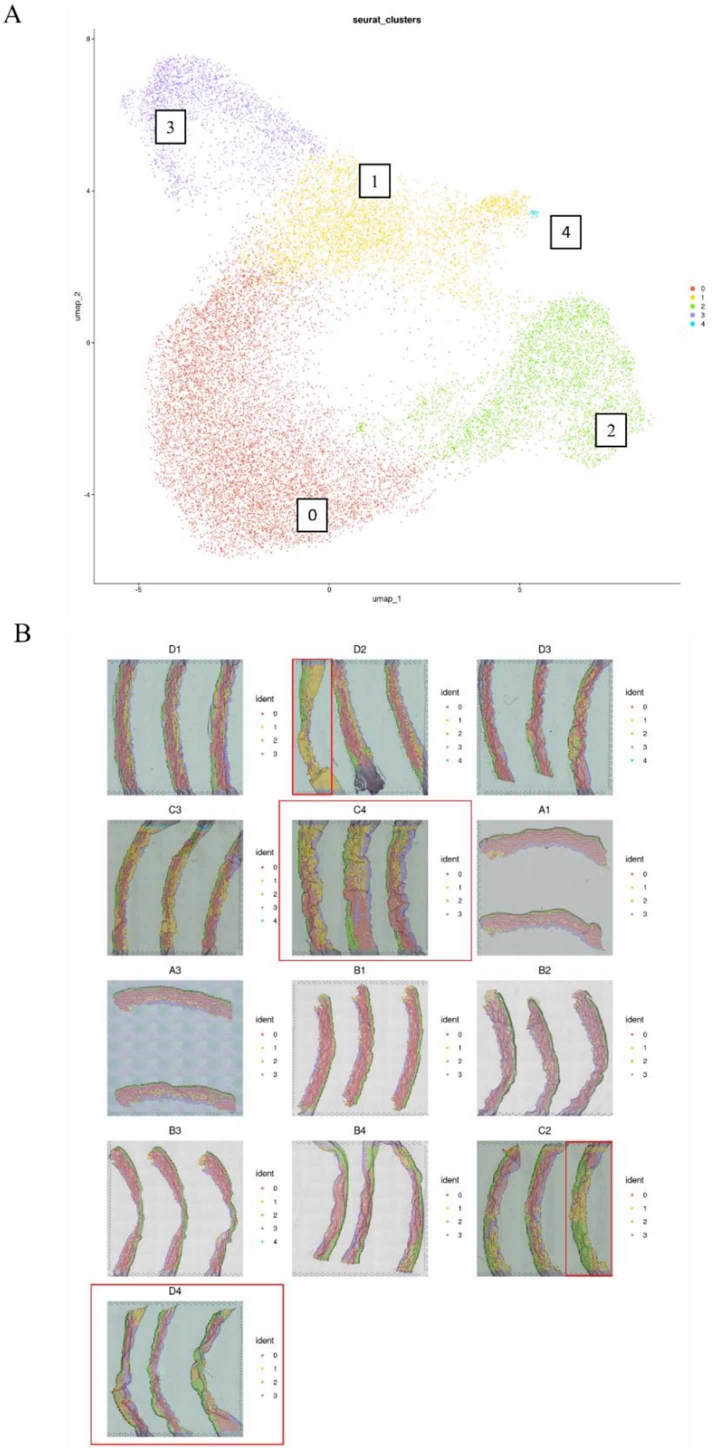

Supplementary Fig. S2. Clustering results before pre-processing. The clustering results were obtained using Seurat pipeline, resolution of 0.2 (A) UMAP for spatial transcriptomics data, showing 4 clusters; (B) Based on the mapping pattern of the clusters to the particular spots in n=13 (8 KTCN corneas, and 5 control corneas), we decided to exclude from analysis the 2 samples and 2 single cryosections marked with red frame, with variability between consecutive sections.

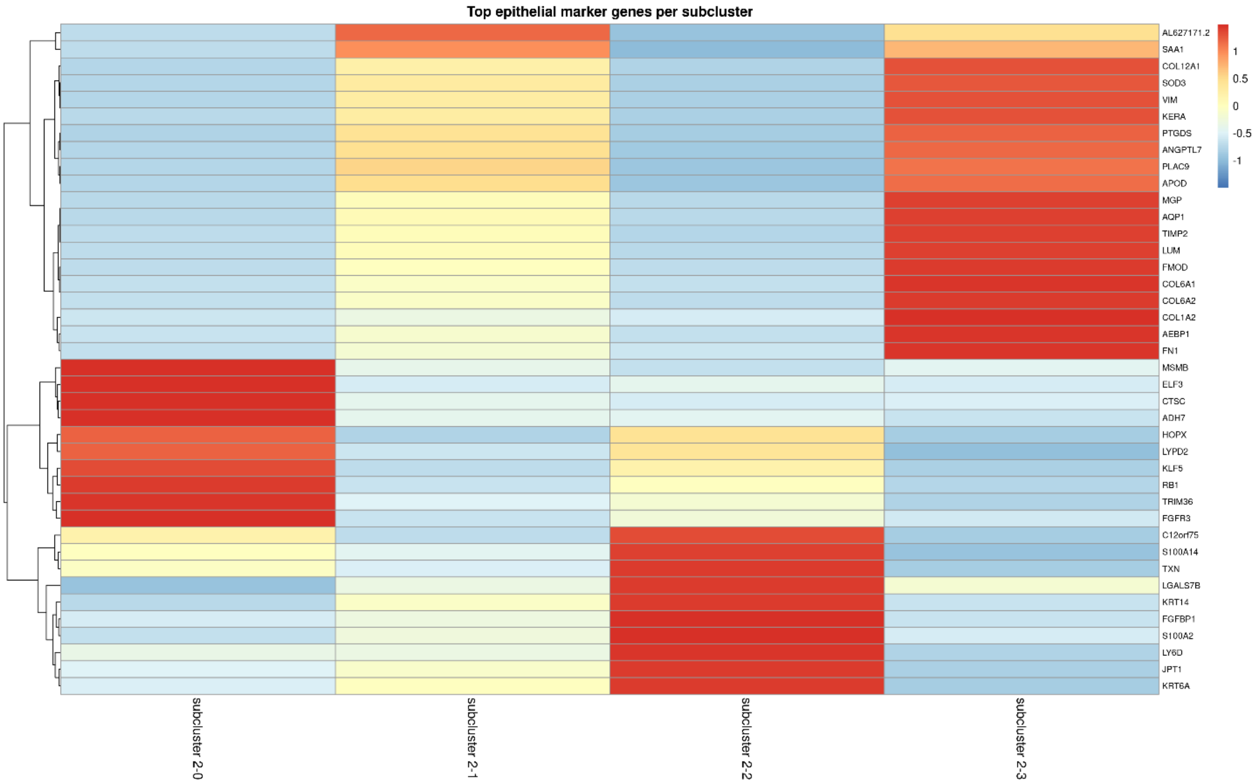

Supplementary Fig. S3.The heatmap of top marker genes for CE subclusters. The red color of bar represents the high gene expression, and the blue color indicates the low gene expression.

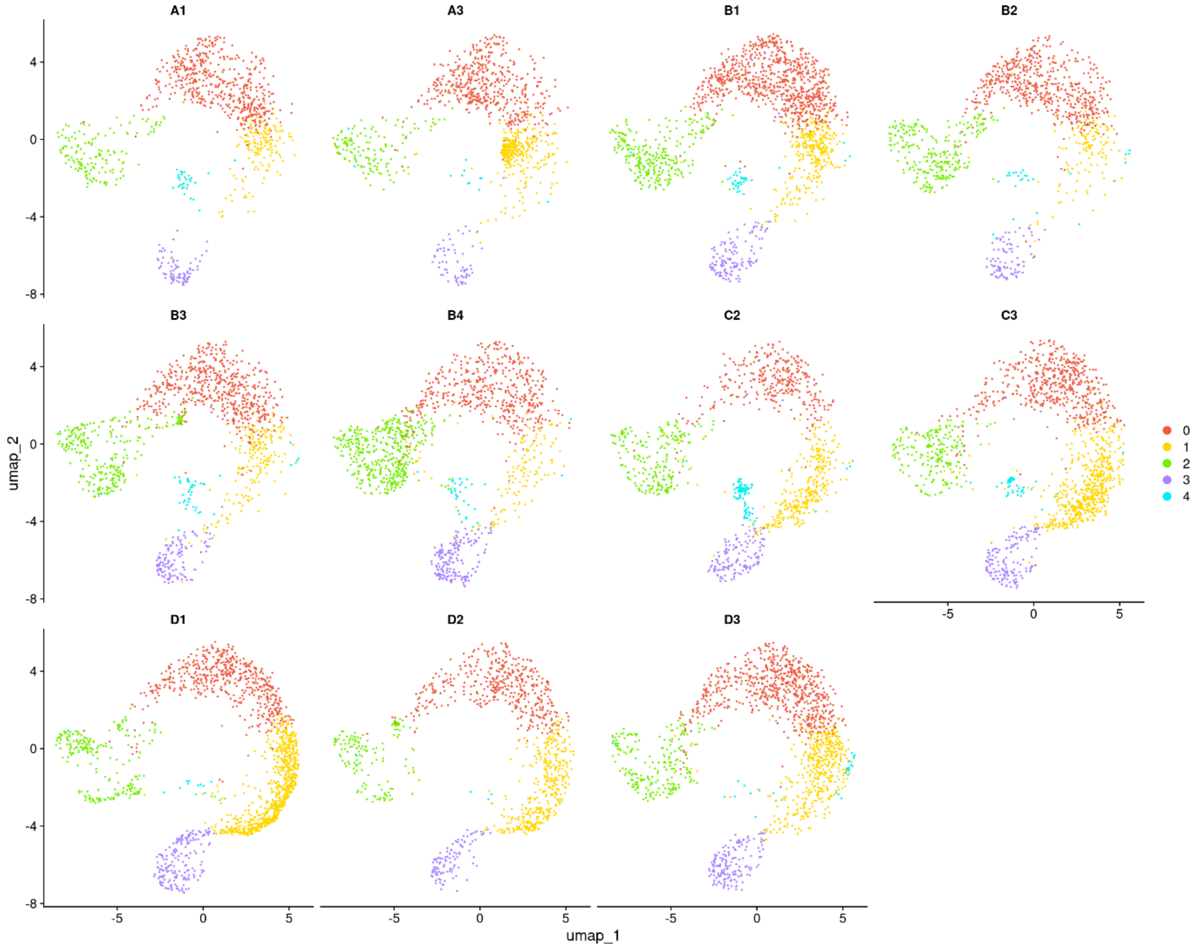

Supplementary Fig. S4.The UMAP visualization for each cornea. Graphically showing the contribution of spots per cluster by each individual. Samples from A1 to C2 represent the KTCN group; C3-D3 control group

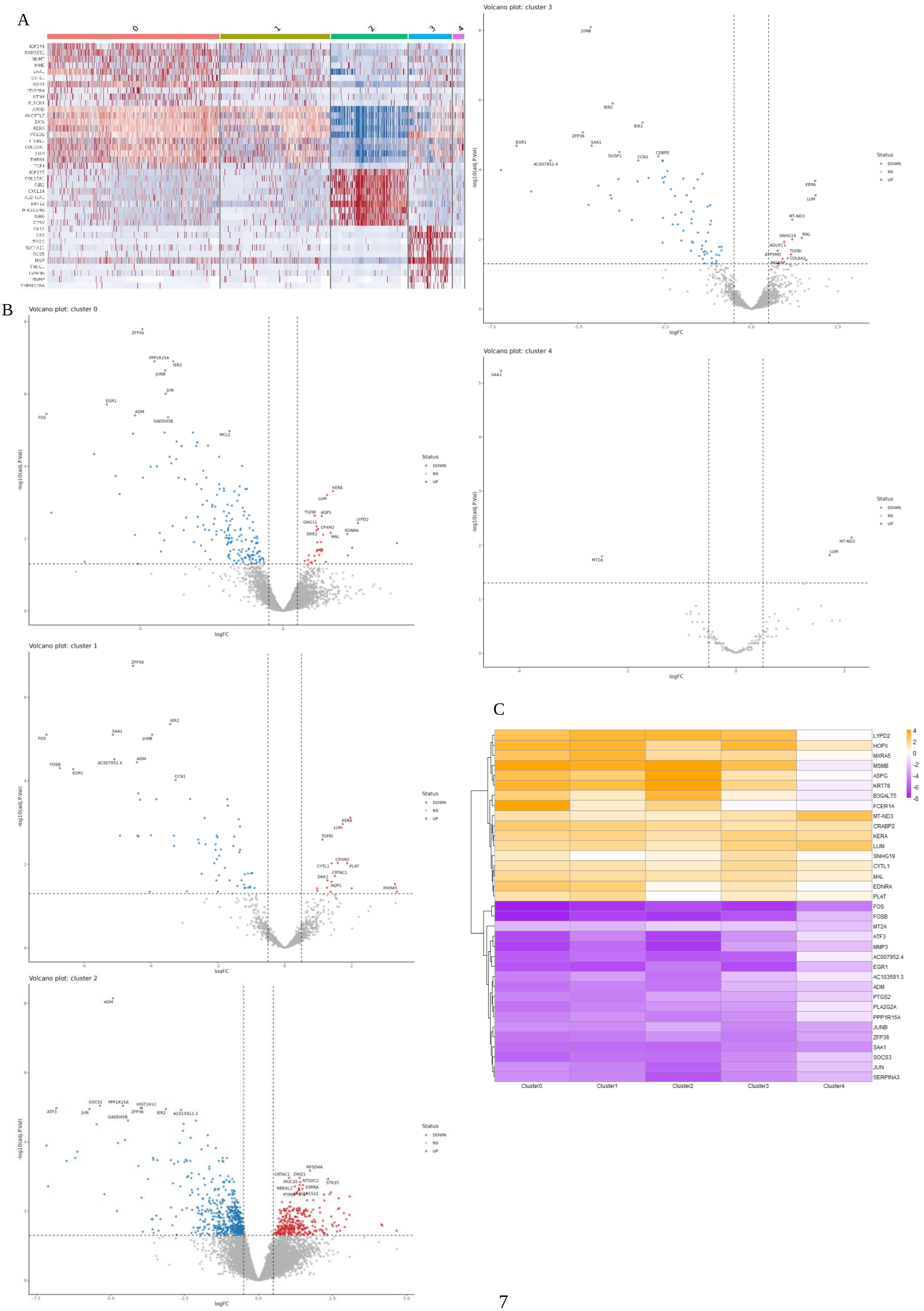
A
0
1
2
3
4
B
Volcano plot: cluster 0
Volcano plot: cluster 1
Volcano plot: cluster 2
Volcano plot: cluster 3
Volcano plot: cluster 4
logFC
-log10(adj.P.Val)
Status
DOWN
NS
UP
C
LYPD2
HOPX
MXRA5
MSMB
ASPG
KRT78
B3GALT5
FCER1A
MT-ND3
CRABP2
KERA
LUM
SNHG19
CYTL1
MAL
EDNRA
PLAT
FOS
FOSB
MT2A
ATF3
MMP3
AC007952.4
EGR1
AC103591.3
ADM
PTGS2
PLA2G2A
PPP1R15A
JUNB
ZFP36
SAA1
SOCS3
JUN
SERPINA3
Cluster0
Cluster1
Cluster2
Cluster3
Cluster4
4
2
0
-2
-4
-6
-8

Supplementary Fig. S5. The results of differentially expressed genes analysis. (A) The heatmap displaying the top 10 differentially expressed genes for each cluster (0-stroma I, 1-stroma II, 2-epithelial, 3-stromal-epithelial, and 4-stroma III) compared with the rest of the data set; red color indicates the high gene expression, whereas blue color indicates low gene expression. The heatmap was prepared without data scaling **(B)** The volcano plots of DEGs for each of the identified clusters (from cluster 0 to cluster 4). The blue dots indicate genes with downregulated expression (adjusted $P < 0.05$ and $\log2FC < -0.5$), and the red dots denote upregulated gene expression (adjusted $P < 0.05$ and $\log2FC > 0.5$) **(C)** The heatmap of the 5 top up- and down-regulated genes for each cluster after DEGs analysis. The genes (rows) were hierarchically clustered using Euclidean distance. Heatmap colors represent log2FC, with a gradient from violet to orange, indicating lower to higher expression levels. n= 11 (n=7 corneas for the KTCN group, and n=4 corneas for the control group).

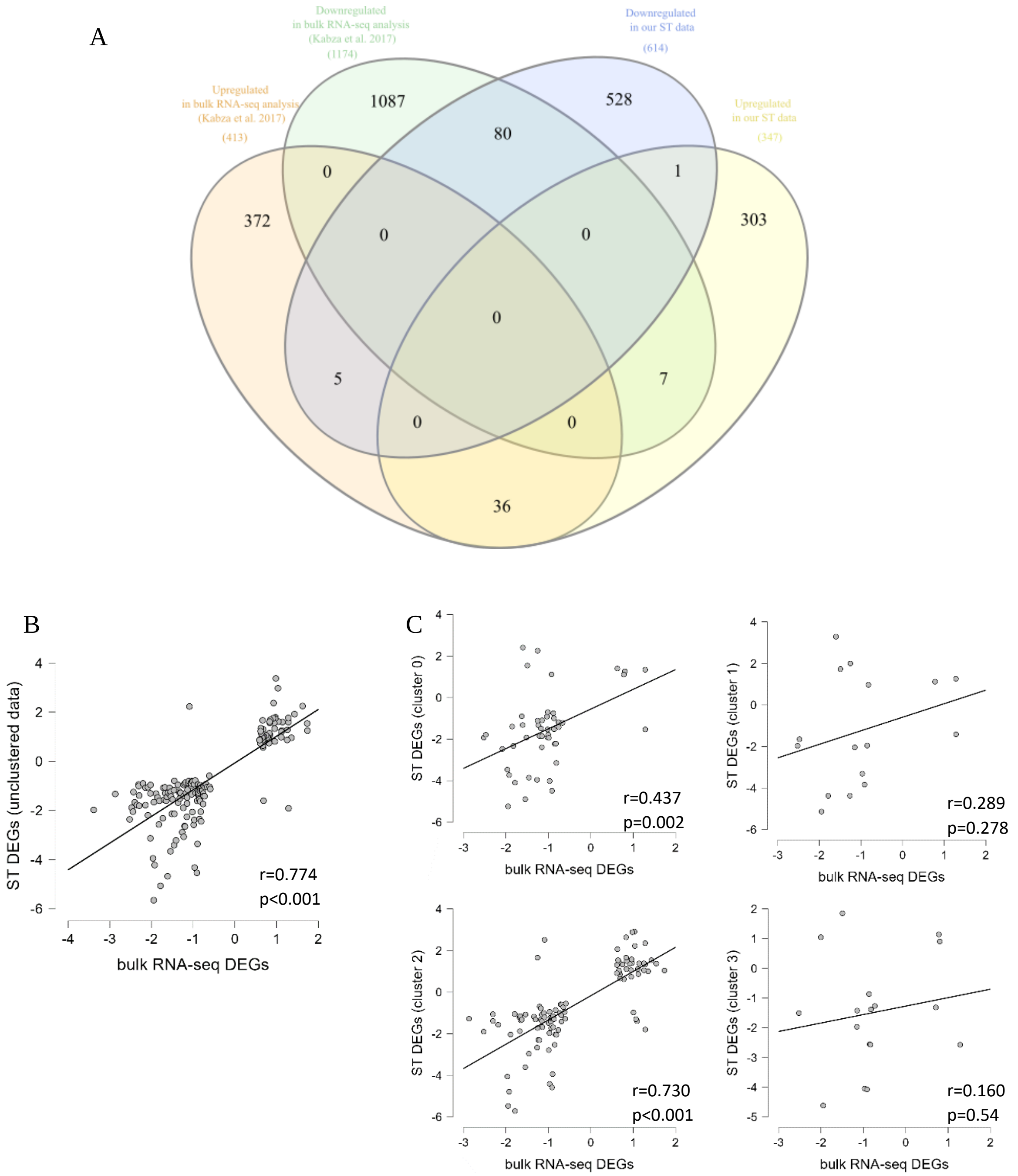

Supplementary Fig. S6. The comparison of ST DEGs outputs with DEGs detected in the bulk RNA-seq approach. (A)The up- and downregulated genes of DEGs analysis from the bulk RNA-seq, n=50 (25 KTCN corneas, and 25 for non-KTCN corneas) as well as up- and downregulated genes derived from our ST approach, n=11 (7 KTCN corneas, and 4 control corneas) were subjected to this comparison. For the bulk RNA-seq analysis, the genes were considered to be differentially expressed if they had a <0.01 FDR and >1.5 fold change. For ST study, the genes meeting the following criteria were considered differentially expressed $0.5 \leq Log2FC \leq -0.5$, p-value < 0.05, and FDR < 0.01; (B) The Pearson's correlation of logarithmized bulk RNA-seq significant DEGs with un-clustered "bulk" ST significant DEGs; (C) The Pearson's correlation of logarithmized bulk RNA-seq DEGs with DEGs of each separate cluster.

A

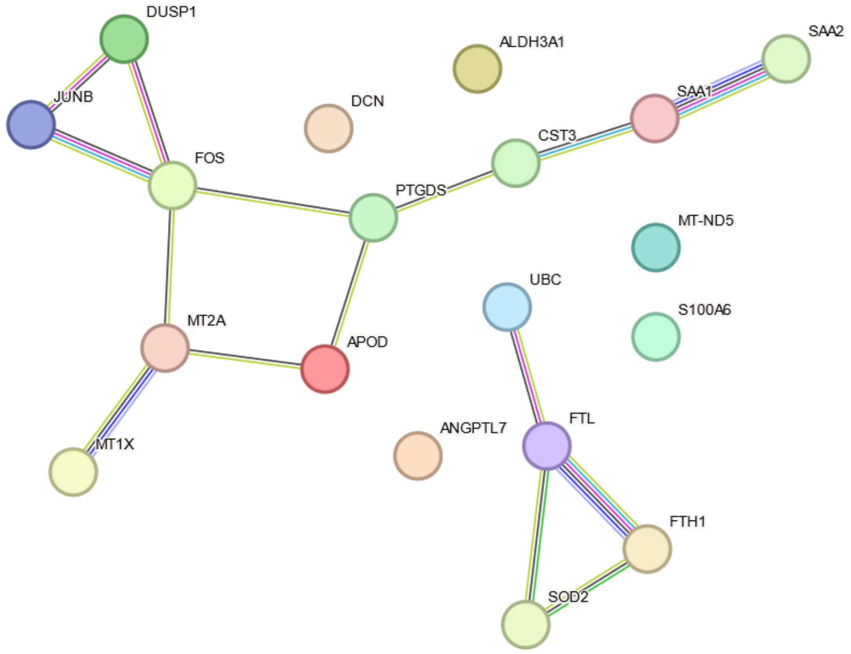

B

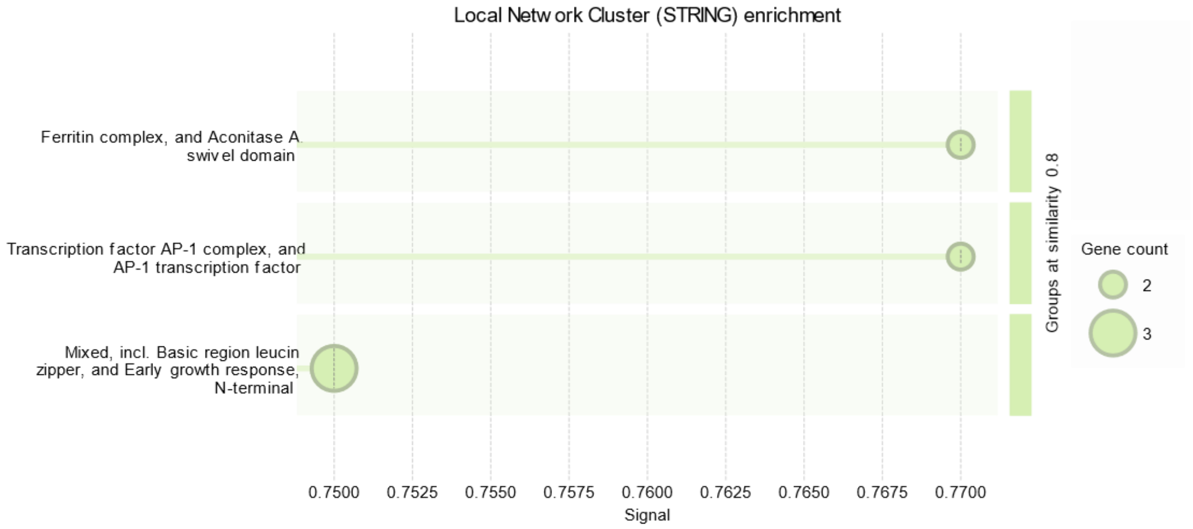

Supplementary Fig. S7 The STRING functional protein association networks analysis. (A) The STRING analysis shows the top genes representing topics specific to control corneas (not detected in KTCN corneas), involved in known and predicted protein-protein interactions. Different coloured lines represent seven types of evidence used to predict associations. Red line: fusion evidence; green line: neighbourhood evidence; blue line: co-occurrence evidence; purple line: experimental evidence; yellow line: text mining evidence; light blue line: database evidence, and black line: coexpression evidence.(B) In the local network cluster (STRING) enrichment analysis the enrichment in the Ferritin complex and AP-1 transcription factor complex was revealed.

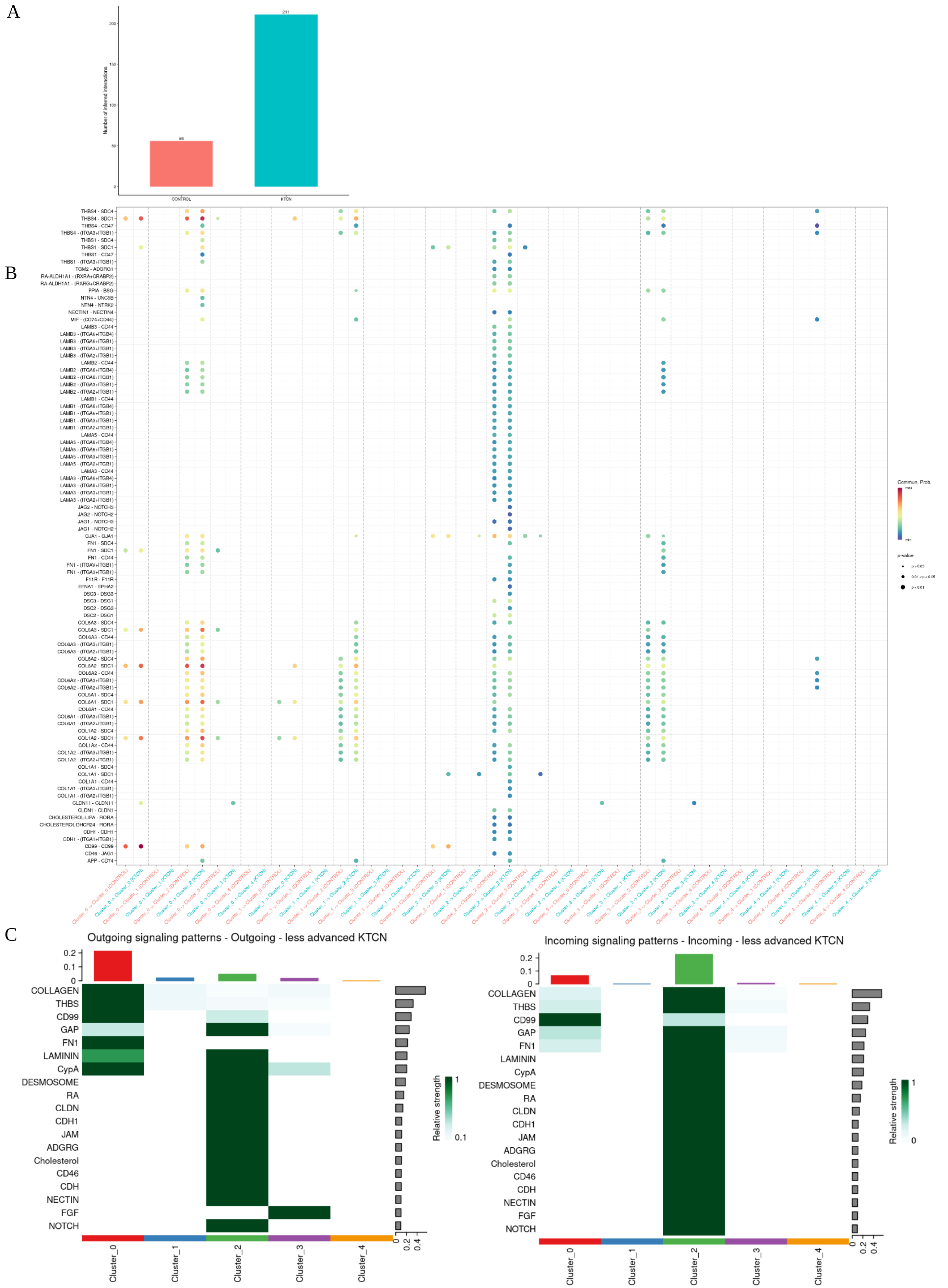

Supplementary Fig. S8 The CellChat analysis of control and KTCN corneas. (A) The overall interaction number for control (red) and KTCN (blue) corneas; (B) The dotplot showing the inferred ligand-receptor pairs for control and KTCN corneas for distinguished pathways; the probability of interaction is marked with a scale of colours from blue – less probable; to red – more probable. The dot's size indicate the p-value. (C) Outgoing (left) and incoming (right) signaling patterns of less advanced KTCN.
