# Supplementary material for: “Eye-Conic” Spatial Transcriptomics Reveals the Layer-Specific Molecular Alterations in Corneas of Patients With Keratoconus
Source: Invest Ophthalmol Vis Sci. 2026 Apr 3;67(4):7. doi: 10.1167/iovs.67.4.7 (PMC13056006; doi:10.1167/iovs.67.4.7)
Supplement: Supplement 3 [file iovs-67-4-7_s003.pdf]

STROBE Statement—Checklist of items that should be included in reports of ***case-control studies***

	Item No	Recommendation
**Title and abstract**	1	(*a*) Indicate the study's design with a commonly used term in the title or the abstract pages 1-2
		(*b*) Provide in the abstract an informative and balanced summary of what was done and what was found pages 2
**Introduction**		
Background/rationale	2	Explain the scientific background and rationale for the investigation being reported pages 3-4
Objectives	3	State specific objectives, including any prespecified hypotheses page 4
**Methods**		
Study design	4	Present key elements of study design early in the paper pages 4-5
Setting	5	Describe the setting, locations, and relevant dates, including periods of recruitment, exposure, follow-up, and data collection pages 4-6
Participants	6	(*a*) Give the eligibility criteria, and the sources and methods of case ascertainment and control selection. Give the rationale for the choice of cases and controls pages 4-7, Supplementary Material: Table S1
		(*b*) For matched studies, give matching criteria and the number of controls per case
Variables	7	Clearly define all outcomes, exposures, predictors, potential confounders, and effect modifiers. Give diagnostic criteria, if applicable
Data sources/ measurement	8*	For each variable of interest, give sources of data and details of methods of assessment (measurement). Describe comparability of assessment methods if there is more than one group
Bias	9	Describe any efforts to address potential sources of bias pages 4-5
Study size	10	Explain how the study size was arrived at pages 4-5
Quantitative variables	11	Explain how quantitative variables were handled in the analyses. If applicable, describe which groupings were chosen and why pages 4-5
Statistical methods	12	(*a*) Describe all statistical methods, including those used to control for confounding pages 5-9
		(*b*) Describe any methods used to examine subgroups and interactions pages 5-9
		(*c*) Explain how missing data were addressed pages 5-9
		(*d*) If applicable, explain how matching of cases and controls was addressed pages 5-9
		(*e*) Describe any sensitivity analyses pages 5-9
**Results**		
Participants	13*	(a) Report numbers of individuals at each stage of study—eg numbers potentially eligible, examined for eligibility, confirmed eligible, included in the study, completing follow-up, and analysed page 9
		(b) Give reasons for non-participation at each stage page 9-13
		(c) Consider use of a flow diagram
Descriptive data	14*	(a) Give characteristics of study participants (eg demographic, clinical, social) and information on exposures and potential confounders
		(b) Indicate number of participants with missing data for each variable of interest
Outcome data	15*	Report numbers in each exposure category, or summary measures of exposure
Main results	16	(*a*) Give unadjusted estimates and, if applicable, confounder-adjusted estimates and their precision (eg, 95% confidence interval). Make clear which confounders were

	adjusted for and why they were included pages 9-13
	(*b*) Report category boundaries when continuous variables were categorized pages 9-13
	(*c*) If relevant, consider translating estimates of relative risk into absolute risk for a meaningful time period pages 9-13

Other analyses	17	Report other analyses done—eg analyses of subgroups and interactions, and sensitivity analyses pages 9-13
**Discussion**		
Key results	18	Summarise key results with reference to study objectives pages 13-18
Limitations	19	Discuss limitations of the study, taking into account sources of potential bias or imprecision. Discuss both direction and magnitude of any potential bias page 18
Interpretation	20	Give a cautious overall interpretation of results considering objectives, limitations, multiplicity of analyses, results from similar studies, and other relevant evidence pages 13-18
Generalisability	21	Discuss the generalisability (external validity) of the study results pages 13-18
**Other information**		
Funding	22	Give the source of funding and the role of the funders for the present study and, if applicable, for the original study on which the present article is based page 1

*Give information separately for cases and controls.

Note: An Explanation and Elaboration article discusses each checklist item and gives methodological background and published examples of transparent reporting. The STROBE checklist is best used in conjunction with this article (freely available on the Web sites of PLoS Medicine at http://www.plosmedicine.org/, Annals of Internal Medicine at http://www.annals.org/, and Epidemiology at http://www.epidem.com/). Information on the STROBE Initiative is available at http://www.strobe-statement.org.
